# Utilizing machine learning for flow zone indicators prediction and hydraulic flow unit classification

**DOI:** 10.1038/s41598-024-54893-1

**Published:** 2024-02-20

**Authors:** Tengku Astsauri, Muhammad Habiburrahman, Ahmed Farid Ibrahim, Yuzhu Wang

**Affiliations:** 1 Department of Petroleum Engineering and Geosciences, King Fahd University of Petroleum & Minerals, 31261 Dhahran, Saudi Arabia; 2 Center for Integrative Petroleum Research, King Fahd University of Petroleum & Minerals, 31261 Dhahran, Saudi Arabia

**Keywords:** Machine learning, Flow zone indicators, Hydraulic flow unit, Reservoir characterization, Energy science and technology, Engineering

## Abstract

Reservoir characterization, essential for understanding subsurface heterogeneity, often faces challenges due to scale-dependent variations. This study addresses this issue by utilizing hydraulic flow unit (HFU) zonation to group rocks with similar petrophysical and flow characteristics. Flow Zone Indicator (FZI), a crucial measure derived from pore throat size, permeability, and porosity, serves as a key parameter, but its determination is time-consuming and expensive. The objective is to employ supervised and unsupervised machine learning to predict FZI and classify the reservoir into distinct HFUs. Unsupervised learning using K-means clustering and supervised algorithms including Random Forest (RF), Extreme Gradient Boosting (XGB), Support Vector Machines (SVM), and Artificial Neural Networks (ANN) were employed. FZI values from RCAL data formed the basis for model training and testing, then the developed models were used to predict FZI in unsampled locations. A methodical approach involves 3 k-fold cross-validation and hyper-parameter tuning, utilizing the random search cross-validation technique over 50 iterations was applied to optimize each model. The four applied algorithms indicate high performance with coefficients determination (R^2^) of 0.89 and 0.91 in training and testing datasets, respectively. RF showed the heist performance with training and testing R^2^ values of 0.957 and 0.908, respectively. Elbow analysis guided the successful clustering of 212 data points into 10 HFUs using k-means clustering and Gaussian mixture techniques. The high-quality reservoir zone was successfully unlocked using the unsupervised technique. It has been discovered that the areas between 2370–2380 feet and 2463–2466 feet are predicted to be high-quality reservoir potential areas, with average FZI values of 500 and 800, consecutively. The application of machine learning in reservoir characterization is deemed highly valuable, offering rapid, cost-effective, and precise results, revolutionizing decision-making in field development compared to conventional methods.

## Introduction

Reservoir characterization is a fundamental part of petroleum engineering that involves gathering and evaluating data to understand the properties of a subsurface reservoir. This process is necessary for making informed decisions involving the production and recovery of hydrocarbons from the reservoir^1^. The information gathered during reservoir characterization is critical for accurate hydrocarbon reserve estimation, optimization of production techniques, risk reduction, and improved recovery, which is vital to financial analysis and decision-making in the oil and gas industry^2^. In addition, reservoir characterization provides valuable information on the reservoir's properties and behavior, which contributes to the development of an optimum field development plan, including the determination of the number and placement of wells, production rates, and field infrastructure design.

Reservoir characterization is a challenging task due to the uncertainty imposed by reservoir heterogeneity, which refers to the variability of reservoir properties across various geological scales. To address this uncertainty, the hydraulic flow unit (HFU) zonation is used to cluster rocks with identical petrophysical and flow characteristics into the same unit^3^. This allows for the prediction of unknown reservoir properties and eliminates unnecessary coring expenses. HFUs are based on geological and physical flow principles and provide a more accurate representation of reservoir heterogeneity compared to traditional lithological or depositional facies-based approaches. The Hydraulic Flow Unit method is related to the Flow Zone Indicator (FZI), a commonly used measure in reservoir characterization. The FZI provides a quantitative method for analyzing the relationship between microscopic characteristics like pore throat size and distribution and macroscopic ones like permeability and porosity. This consequently suggests that rock properties derived from depositional and diagenetic processes play a significant role in determining the surface area, shape factor, and tortuosity of carbonates, and thus the FZI value^4^.

Conventional methods for reservoir characterization primarily focus on directly measuring or estimating permeability and porosity, which are crucial for understanding reservoir potential. The primary tools for this purpose are core measurements and well logs. Core measurements involve physically extracting a sample from the reservoir and analyzing it to determine properties like permeability and porosity. Well logs, on the other hand, are continuous recordings of various physical parameters along the wellbore, providing indirect estimates of these reservoir properties.

While core measurements offer high accuracy, they are often expensive, time-consuming, and only provide data for a limited section of the reservoir. Well logs, including tools like bulk density, neutron porosity, sonic, and nuclear magnetic resonance logs, are more extensive but can sometimes yield less satisfactory results. This is due to uncertainties in the empirical parameters used for interpretation and the adaptability issues of response equations to different reservoir conditions. These limitations of conventional methods highlight the need for more efficient and comprehensive approaches in reservoir characterization (Rock Typing)^5,6^. Therefore, there is a need to identify advanced methods capable of overcoming the limitations inherent in traditional reservoir characterization techniques.

AI and Machine Learning (ML) offer solutions to these challenges by efficiently processing vast quantities of data, surpassing the limitations of human analysis in both speed and complexity. These advanced technologies can interpret intricate datasets from logs more effectively, identifying patterns and correlations that might be missed by traditional methods. Furthermore, AI-driven methods are not confined to the data from cored intervals, enabling a more comprehensive analysis of the reservoir. This holistic approach can integrate diverse data sources, including seismic, geological, and production data, offering a more nuanced understanding of reservoir characteristics. Studies have utilized a range of supervised machine learning algorithms, including Random Forest (RF)^7^, Support Vector Machines (SVM)^8^, Artificial Neural Networks (ANN)^9^, adaptive network fuzzy inference system (ANFIS)^10^, and Extreme Gradient Boosting (XGB)^6^, to accurately predict permeability values. Additionally, unsupervised machine learning algorithms such as K-Means have been studied to classify the reservoir based on the hydraulic flow units (HFUs)^11,12^.

The main objective of this study is to create a supervised machine-learning model that directly estimates the flow zone indicator (FZI) at unsampled locations using well-logging data during the initial exploration phase. This approach is highly valuable as it allows for the direct determination of FZI at specific depths of interest, leveraging the power of supervised machine learning. Additionally, an unsupervised machine-learning model will be developed to cluster hydraulic flow unit numbers in the target zone. This clustering approach is also valuable as it enables the assessment of distinct petrophysical properties associated with flow units, which greatly influences reservoir characterization.

To accomplish the study's objective, the implementation of popular machine learning algorithms like K-Means for the unsupervised machine learning model, and Random Forest, Extreme Gradient Boosting, Support Vector Machines, and Artificial Neural Network for the supervised machine learning model is planned. Additionally, the credibility of the results will be ensured by evaluating the physics-based approach in conjunction with the data-driven approach of supervised machine learning. This combination of approaches will enhance the classification of rock reservoir types, resulting in improved accuracy and efficiency. Therefore, this research aims to introduce a dependable and data-driven approach for predicting flow zone indicators in unsampled locations, utilizing advanced machine learning techniques. This innovative methodology is poised to significantly contribute to the advancement of rock reservoir type classification within the petroleum industry, marking a shift towards more sophisticated, analytics-based strategies.

## The flow zone indicator (FZI)

In reservoir characterization, predicting permeability is crucial for understanding hydrocarbon production. The Hydraulic Flow Unit (HFU) approach was first introduced by^3^ which is based on the modification of the Kozeny-Carman equation:

1 $$k=\left(\frac{1}{{K}_{T}{S}_{{v}_{gr}}^{2}}\right)\times \left(\frac{{\phi }_{e}^{3}}{{\left(1-{\phi }_{e}\right)}^{2}}\right)$$

where $k$ is permeability in m^2^, ${\phi }_{e}$ is effective porosity, ${K}_{T}$ is the pore-level effective zoning factor and ${S}_{{v}_{gr}}$ is the specific surface area per unit grain volume. The ${K}_{T}$ parameter is a function of pore size and shape, grain size and shape, pore and grain distribution, tortuosity, cementation, and pore system (intergranular, intracrystalline, vuggy, or fractured)^13^.

The HFU approach uses the normalized porosity index or the void ratio (${\phi }_{z}$) and reservoir quality index (RQI) to predict permeability. The method involves plotting ${\phi }_{z}$ against RQI on a log–log scale and fitting a unit slope trend line. The Flow Zone Indicator (FZI), which characterizes the geological and petrophysical attributes of a given HFU, is determined by the intercept value of the trend line at (${\phi }_{z}$) = 1. The previous parameters are calculated using the following equations.

2 $$RQI=0.0314\sqrt{\frac{k}{\phi }}$$

3 $${\phi }_{z}=\frac{{\phi }_{e}}{1-{\phi }_{e}}$$

4 $$FZI=\frac{1}{\sqrt{{F}_{S}}\tau {S}_{gv}}=\frac{RQI}{{\phi }_{z}}$$

where $k$ is permeability in mD, ${\phi }_{e}$ is effective porosity in fractions, ${F}_{s}$ is the shape factor, $\tau$ is the tortuosity, ${S}_{gv}$ is the surface area per unit grain in μm. The permeability can be recalculated based on the flow unit of a sample, considering the FZI and effective porosity, using the following equation.

5 $$k=1014.24{\left(FZI\right)}^{2}\frac{{\phi }_{e}^{3}}{{\left(1-{\phi }_{e}\right)}^{2}}$$

When the samples for a given HFU are closely aligned with the trend line, the FZI value is equal to or close to the FZI arithmetic average of these samples, and the predicted permeability is identical to the measured one. However, if the samples are scattered around the trend line, the FZI value differs greatly from the FZI arithmetic average, and the predicted permeability is far from the measured one, with a significant error^14^. Fine-grained rocks, poorly sorted sands, rocks with authigenic pore filling, pore filling, and pore bridging clays are more likely to have a large surface area and a high degree of tortuosity, as stated by^3^. The shape factors and tortuosity of coarse-grained, well-sorted sands are much lower. Integrating FZI with other well logs and core data enables the classification of HFUs, leading to more accurate reservoir characterization and better reservoir management.

## Machine learning description

### Supervised machine learning

#### Random forest (RF)

Random forest is implemented via bootstrap aggregation^15^. The bagging is based on the concept of building multiple decision trees independently from one another using a subset of the input predictor parameters and a bootstrap sample of the training data^16^. It randomly selects the training dataset **T**b (b = 1, …, **B**) from the whole training set **T** with replacement (bootstrapping sampling) and randomly selects M features or input variables from P input variables or (M < P)^17,18^. By following these steps, the proxy model's bias, excess variance, and overfitting will be reduced to acceptable levels. Like decision trees, random forests are effective at resolving non-linear patterns within data while also being scalable and resistant to outliers in imbalanced datasets^19–23^.

For each tree within the Classification and Regression Tree (CART) framework, the ideal division is calculated using a random selection of both **T**b and P features. The collective set of these trees can be expressed as an ensemble.

6 $$\left\{{\phi }_{{T}_{b},m}\mid b=1,\cdots ,B\right\}$$

In the regression approach used by the Random Forest algorithm, the final prediction is derived through an averaging process rather than majority voting. The prediction $\widehat{Y}$ for a given input *X* is calculated as the average of the predictions from all the individual trees in the ensemble:

7 $$\widehat{\mathbf{Y}}={\phi }_{T,P}(\mathbf{X})=\frac{1}{B}\sum_{b=1}^{B} {\phi }_{{T}_{b},m}(\mathbf{X})$$

This equation suggests that the collective prediction is the meaning of the outcomes from each of the **B** individual Classification and Regression Trees (CART) that constitute the forest. By averaging, Random Forest harnesses the diversity of the ensemble, effectively reducing the overall prediction error. This method capitalizes on the ensemble's ability to minimize the average squared error across the predictions, often resulting in a more accurate prediction than any single tree's output^18,23^.

#### Extreme gradient boosting (XGB)

The gradient boosting approach is a robust ensemble training algorithm designed for both non-linear classification and regression applications by upgrading a weak learning model into a strong learner^24–26^. The primary objective of the gradient boosting approach is to identify a new sub-model with a lower error rate than the previous model. Hence, this method relies on the use of multiple models (bagging) which are trained to minimize errors from the previous method^17,27^.

One of the most well-known gradient-boosting enhancements is Extreme Gradient Boosting (XGB), which employs Gradient Boosting Decision Trees (GDBT)^28^. This method avoids overfitting because it considers more regularization terms than standard gradient tree boosting. Furthermore, it enhances model robustness by employing sampling techniques across both rows and columns, effectively diminishing the model's variance^29^. A key factor in XGBoost's effectiveness is its ability to scale efficiently across various configurations. The ensemble model of XGBoost is formulated in an additive manner.

8 $$\widehat{{y}_{i}}=\sum_{K=1}^{K} {f}_{K}\left({x}_{i}\right),{f}_{K}\in F$$

where $f$ symbolizes a specific tree within the space $F$, which encompasses the entire set of regression trees. Here ${x}_{i}$, ignifies the $i$-th eigenvector, and $K$ is the total count of trees in the model. The expression of cost function presented as follows:

9 $${L}_{(j)}=\sum_{i} l\left({y}_{i},{\widehat{y}}_{i}\right)+\sum_{K}\Omega \left({f}_{K}\right)$$

where The sum of the loss function $l\left({y}_{i},{\widehat{y}}_{i}\right)$, measuring the difference between the observed ${y}_{i}$ and the predicted ${\widehat{y}}_{i}$ values and $\Omega$ denotes the regular punishment. The regularization term $\Omega$ itself is further defined as a combination of two components.

10 $${\Omega }_{\left({f}_{K}\right)}=\gamma T+\frac{1}{2}\lambda {\omega }^{2}$$

$\gamma T$, where $\gamma$ is the coefficient penalizing the complexity of the model by the number of leaf nodes $T$, enforcing the ${\mathcal{l}}_{1}$ norm, while $\frac{1}{2}\lambda {\omega }^{2}$, with $\lambda$ as the coefficient for the ${\mathcal{l}}_{2}$ norm and $\omega$ as the leaf weight.

#### Support vector machines (SVM)

Support vector machines are based on the inductive concept of structural risk minimization (SRM), which allows for reasonable generalizations to be made from a limited set of training examples^30–33^. This method utilizes a margin-based loss function to control the input space dimensions and a kernel function to project the prediction model onto a higher-dimensional space.

A support vector regressor (SVR) is a member of the Support Vector Machine, which has extremely potent and flexible performance, is not confined to linear models, and is resistant to outliers. This method utilizes the kernel trick to translate the original data into a higher-dimensional space without explicitly declaring the higher dimension^34,35^. This method's compatibility with linear models (using linear kernels) or non-linear models (using polynomial or radial kernels) makes it extremely versatile^17^. The effectiveness of the SVR relies heavily on the model selection and kernel function settings (C, Gamma, and Epsilon)^36^.

The introduction of Vapnik's epsilon-insensitive loss function has enabled Support Vector Regression (SVR) to effectively address nonlinear regression estimation challenges. This approach involves the approximation of given datasets using this specialized loss function.

10 $$D=\left\{\left({x}^{1},{y}^{1}\right),\dots ,\left({x}^{l},{y}^{l}\right)\right\},x\in {R}^{n},y\in R$$

With a linear function

11 $$f(\mathbf{x})=\langle {\varvec{\omega}},\mathbf{x}\rangle +b,{\varvec{\omega}}\in X,b\in R$$

where the dot product in X is denoted by $\langle ,\rangle$. SVR aims to find a function $f(\mathbf{x})$ that approximates output values within a deviation of $\in$ from the actual training data. The choice of $\varepsilon$ is crucial, as smaller values lead to tighter models that penalize a larger portion of the training data, while larger values result in looser models with less penalization. The ideal regression function is identified by addressing an optimization problem, which is designed to calculate the values of ${\varvec{\omega}}$ and $b$:

12 $${\text{minimize}}\frac{1}{2}\parallel{\varvec{\omega}}{\parallel }^{2}+C\sum_{i=1}^{l} \left({\xi }_{i}+{\xi }_{i}^{*}\right)$$

13 $$\text{subject to }\left\{\begin{array}{l}{y}_{i}-\langle {\varvec{\omega}},{\mathbf{x}}_{i}\rangle -b\le \varepsilon +{\xi }_{i}\\ \langle {\varvec{\omega}},{\mathbf{x}}_{i}\rangle +b-{y}_{i}\le \varepsilon +{\xi }_{i}^{*}\\ {\xi }_{i},{\xi }_{i}^{*}\ge 0\end{array}\right.$$

where ${\xi }_{i}$ and ${\xi }_{i}^{*}$ are the slack variables, and the model parameters ${\varvec{\omega}}$ and $b$. This approach balances minimizing training error and penalizing model complexity, thus controlling the generalization error. The regularization constant $C$ in the optimization formulation helps to trade off between these two aspects. The epsilon-insensitive loss function further adds to this balance by penalizing errors only when they exceed $\varepsilon$. This methodology allows SVR to achieve better generalization performance compared to some other models like neural networks^37^.

#### Artificial neural network (multi-layer perceptron)

Artificial Neural Network (ANN) or multi-layer perceptron is one of the most effective machine learning approaches. Its mathematical design is inspired by biological neural networks. This technique consists of 3 main layers, the input layer is aimed for receiving input information from X variable. This data will be received and learned by the hidden layer. This information will be generated by the output layer as a consequence of testing^38–41^.

This study will concentrate on feed-forward back-propagation neural networks, one of the numerous forms of neural networks. In this method, the input information flows in a forward manner from the input layer to the hidden layer and ends up in the output layer. The errors that arise during this procedure will be calculated and backpropagated by resetting the network's weight and bias. It is an iterative procedure until the finest inaccuracy is discovered^34,41,42^.

Hagan and colleagues^41^ stated that single cycle of the process is described by the following equation.

14 $${Z}_{k+1}={Z}_{k}-{\alpha }_{k}{g}_{k}$$

where ${g}_{k}$ represents the current gradiend, ${Z}_{k}$ denotes the current set of weights and biases, and ${\alpha }_{k}$ is the learning rate. To adjust the connection weights for a specific neuron $i$ during a particular iteration $p$, the following equation outlines the process^43^.

15 $${w}_{i}(p+1)={w}_{i}(p)+\Delta {w}_{i}(p)$$

This equation updates the weight of the $i$-th neuron for the next iteration $(p+1)$ by adding a weight correction factor $\Delta {w}_{i}(p)$ to the current weight ${w}_{i}(p)$.

The weight correction factor $\Delta {w}_{i}(p)$ is calculated based on the equation.

16 $$\Delta {w}_{i}(p)=\alpha {x}_{i}(p)e(p)$$

For the $j$-th neuron in a hidden layer ${\gamma }_{i}$, alternate expression for the weight correction factor $\Delta {w}_{i}(p)$ is defined as follows^43,44^.

17 $${w}_{i}(p)=\alpha {\gamma }_{i}(p){\delta }_{k}(p)$$

where ${\delta }_{k}(p)$ denotes the error gradient at neuron $k$ in the output layer during the iteration $p$. This equation is commonly referred to as the delta rule.

### Unsupervised machine learning

#### K-means clustering

In this study, the K-Means algorithm is exclusively used as an unsupervised machine learning technique. It is selected for its simplicity and widespread application in clustering tasks. The algorithm minimizes a performance criterion called P, which is calculated as the sum of squared error distances between data points and their corresponding cluster centers^45^. The algorithm begins with a random initial partition, and patterns are then reassigned to clusters based on their similarity to the cluster centers until a convergence criterion is satisfied, such as no further reassignments or a significant reduction in squared error after a certain number of iterations^46^. The squared error for a clustering $L$ of a pattern set $H$ containing $K$ clusters is as follows.

18 $${e}^{2}\left(H,L\right)=\sum_{j=1}^{K}\sum_{i=1}^{{n}_{j}}{\Vert {x}_{i}^{j}-{c}_{j}\Vert }^{2}$$

where ${x}_{i}^{j}$ is the ith pattern belonging to the jth cluster and ${c}_{j}$ is the centroid of the jth cluster.

In this study, along with the K-means algorithm, the Gaussian Mixture Model will also be implemented to reinforce the confidence in the outcomes derived from the K-means clustering. The Gaussian Mixture Model (GMM) offers a probabilistic approach to clustering, presenting the advantage of accommodating clusters of different sizes and orientations due to its use of covariance matrices. This capability enables the GMM to identify and adapt to elliptical or anisotropic clusters, unlike simpler algorithms like k-means which assume isotropic clusters. Additionally, GMM provides a soft-clustering approach, assigning probabilities of membership to each point for all clusters, rather than forcing a hard assignment. This results in a more nuanced understanding of the data's structure, particularly useful when the relationship between variables is complex and not easily separable into distinct groupings^47^. Hence, incorporating both K-means and Gaussian Mixture Model (GMM) methods in a single study leverages the strengths of both clustering techniques.

## Methodology

### Data acquisition

This study analyzes open-source data consisting of thousands of well logs from the Halibut Oil Field, which are supplemented with routine core analysis studies. A total of 212 data sets are chosen for analysis, based on the specific formation depth and the availability of porosity and permeability data at that depth. These data sets encompass 17 different types of well-log information, including Corrected Gamma Ray (CGR), Bulk Density Correction (DRHO), Delta-T Compressional (DT5), Gamma Ray (GR), High-Resolution Enhanced Thermal Neutron (HNPO), Laterolog Deep Resistivity (LLD), High-Resolution Laterolog Resistivity (LLHR), Laterolog Shallow Resistivity (LLS), Mud Resistivity (MRES), Micro Spherically Focused Resistivity (MSFC), Thermal Neutron Porosity (NPHI), Enhanced Thermal Neutron Porosity (NPOR), Potassium Concentration (POTA), Bulk Density (RHOB), Spontaneous Potential (SP), Thorium Concentration (THOR), and Uranium Concentration (URAN), alongside porosity and permeability data. The focus of this study is the FZI parameter, which is directly influenced by permeability.

**Figure 1:**
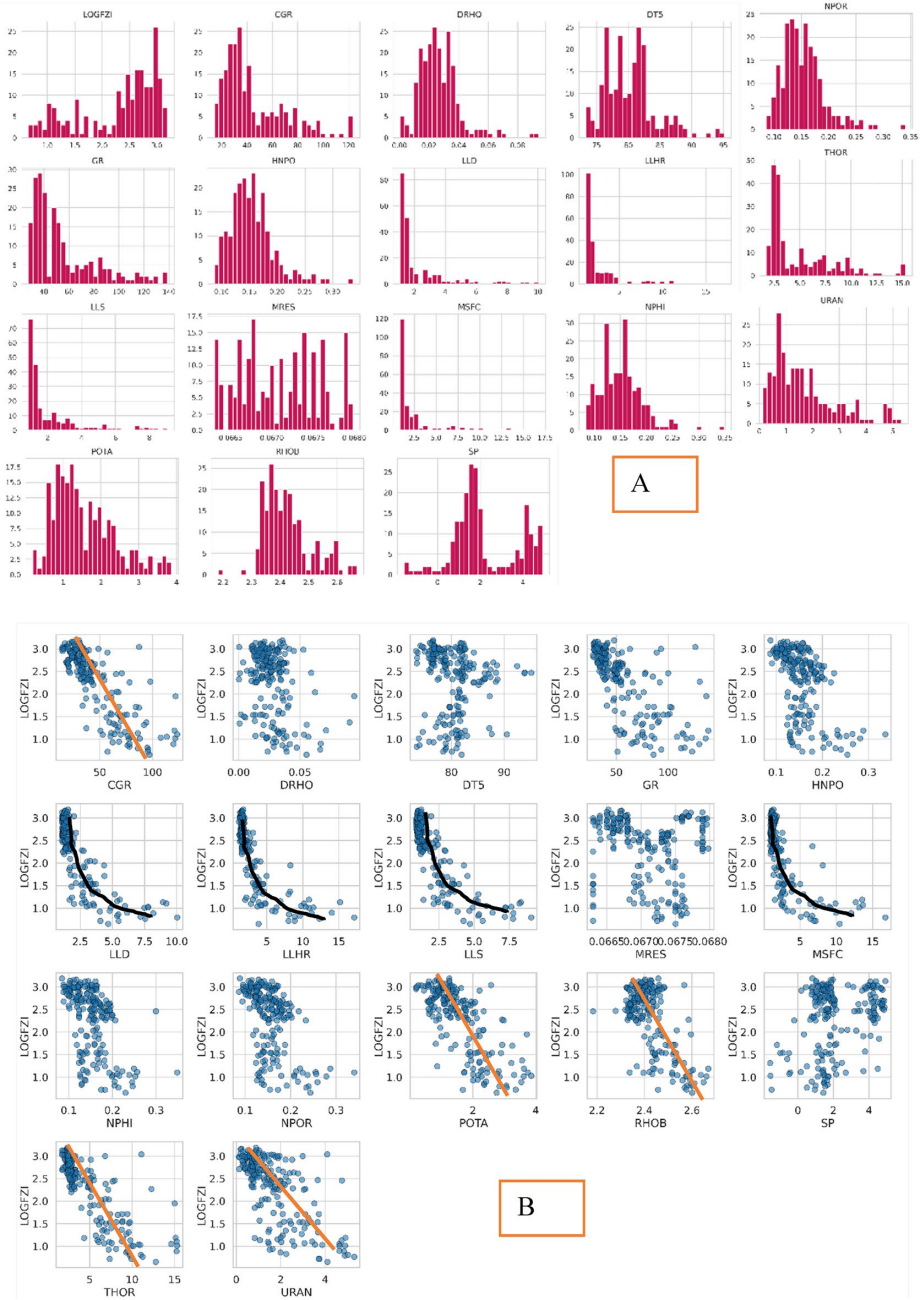
Histogram of 17 well-log parameters, illustrating the diverse distribution types for each parameter. The LOGFZI distribution demonstrates a closer resemblance to a log-normal distribution compared to the original FZI distribution.

It is acknowledged that the FZI exhibits a non-normal distribution, as evident from Fig. 1. Consequently, predicting the FZI directly could potentially lead to misleading results due to its extremely non-normal distribution. To address this issue, an approach is taken to transform the FZI values using a logarithmic scale, aiming to approximate a normal distribution, as illustrated in Fig. 1. To provide an initial understanding of the data, Table 1 presents the data statistics, while Fig. 1 showcases the distribution of each parameter considered in the study and a pair chart for the input versus the output parameter. The cross plot between the input and the output parameters in Fig. 1b shows a linear (in orange) and nonlinear (in black) relationship between the output and input parameters.

**Table 1:** Statistical summary of 17 well-log parameters, including FZI and LOGFZI.

No.	Parameter	Count	Mean	Standard deviation	Minimal	Maximal
1	LOGFZI	212	2.31	0.71	0.65	3.19
2	FZI	212	456.23	400.79	4.50	1536.15
3	CGR	212	45.03	24.70	14.16	123.35
4	DRHO	212	0.03	0.01	0.00	0.09
5	DT5	212	80.31	3.97	73.31	95.14
6	GR	212	55.62	26.47	27.68	138.58
7	HNPO	212	0.15	0.04	0.09	0.34
8	LLD	212	2.20	1.69	1.08	10.04
9	LLHR	212	2.91	2.73	1.15	17.12
10	LLS	212	2.08	1.67	0.88	9.04
11	MRES	212	0.07	0.00	0.07	0.07
12	MSFC	212	2.45	2.72	0.87	16.75
13	NPHI	212	0.15	0.04	0.08	0.35
14	NPOR	212	0.15	0.04	0.09	0.34
15	POTA	212	1.55	0.80	0.19	3.86
16	RHOB	212	2.43	0.08	2.18	2.67
17	SP	212	2.18	1.51	− 1.56	4.92
18	THOR	212	4.83	3.15	1.75	15.29
19	URAN	212	1.66	1.20	0.12	5.32

**Figure 2:**
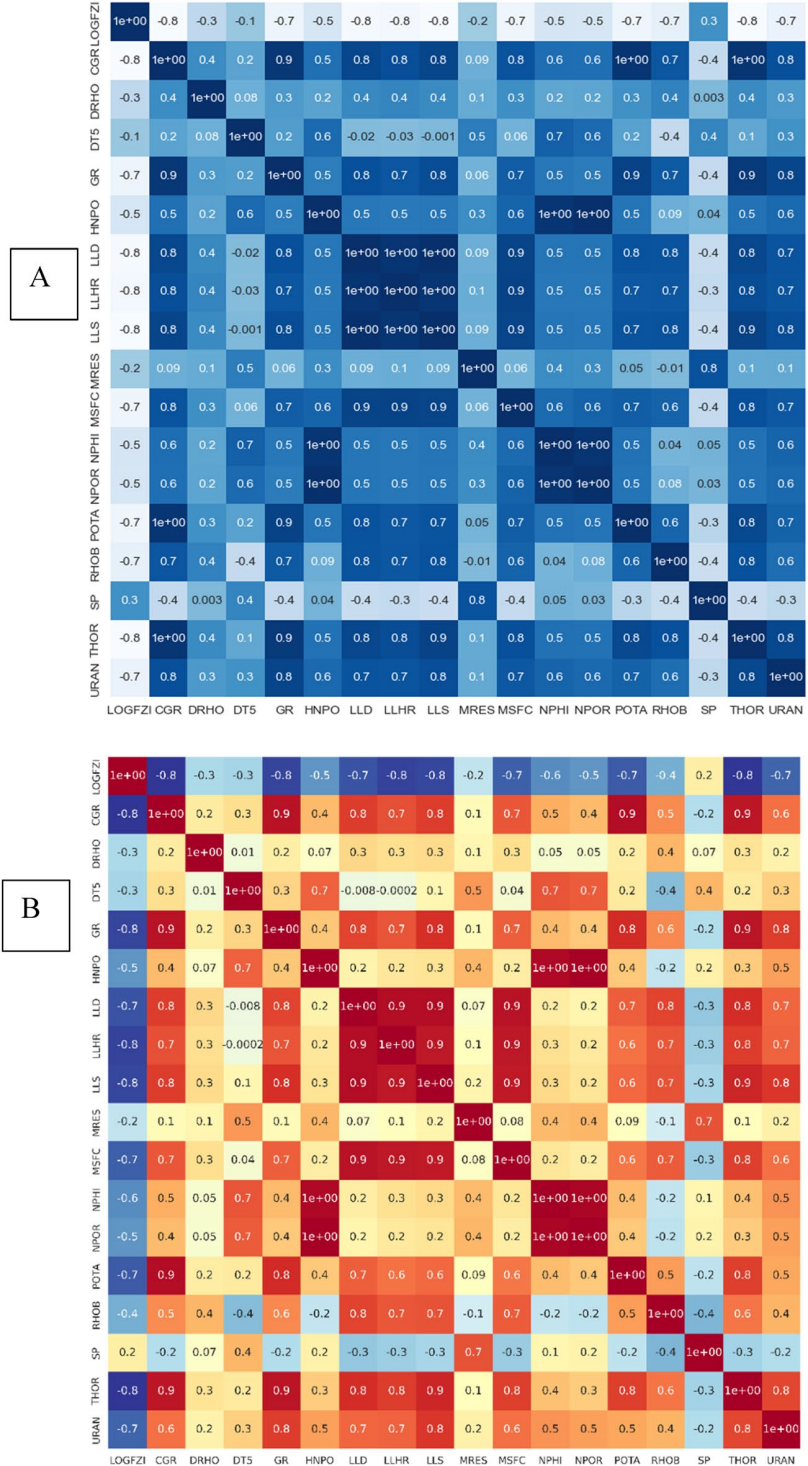
Heatmap of correlation coefficients between each parameter, illustrating the strength of correlation for all parameters; (**A**) Pearson’s Coefficients, (**B**) Spearman’s coefficients. The LOGFZI exhibits noticeable strong correlations (mostly negative) with several parameters, (Heat map was generated using seaborn. heatmap python library).

Eighteen parameters (including the LOGFZI) were chosen at the initial phase of this study, as shown in Table 1. It is necessary to reduce the number of parameters to optimize the model's dimensionality and improve its processing time^48^. However, initially applying all 18 input factors will allow for a more comprehensive understanding of how these parameters affect the precision with which the machine learning model predicts the flow zone indicator. When the connection between input factors and model accuracy is better understood, it's possible to reduce the number of parameters and thereby boost model efficiency. The correlation coefficient analysis of each input parameter to the output parameter of LOGFZI is presented in Fig. 2. The heat map was generated using seaborn.heatmap python library. Figure 2a presents Pearson’s correlation coefficients, that highlight the linear relationship between the parameters with each other, while Fig. 2b presents the Spearman’s correlation coefficients, that was used to exclude the nonlinearity and outliers’ effect. The correlation coefficients for most parameters remained consistent, except in a few instances where the correlation either increased or decreased when Spearman's coefficient was calculated compared to Pearson’s coefficients. This variation can be attributed to the presence of outliers or nonlinear relationships. For instance, the correlation for DTS slightly increased from -0.1 to -0.3, indicating a more negative relation with LogFZI. Similarly, the LLD coefficient increased from − 0.7 to − 0.8 due to the nonlinear relation between LogFZI and LLD. Conversely, the correlation for RHOB decreased from − 0.7 to − 0.4.

Data normalization needs to be done to improve integrity and reduce data redundancy especially for the algorithm that basically relies on the distance technique (KNN and SVR). This is normally done because the input and output data used in the study have very large unit and range differences. The normalization technique employed in this study is the MinMaxScaler. A significant benefit of this scaler is its capability to preserve the original shape of the dataset's distribution. This preservation is critical as it ensures the integral information within the data remains unaffected during scaling. Unlike several other scaling methods, MinMaxScaler does not alter the core characteristics of the original data, thus maintaining the crucial details and patterns necessary for accurate analysis. The normalization formula applied is:

19 $${x}_{{\text{norm}}}= \frac{x- {x}_{{\text{min}}}}{{x}_{{\text{max}}}- {x}_{{\text{min}}}}$$

where ${x}_{{\text{norm}}}$ is a normalized value with a range of values 0–1, x is the variable on the dataset while max and min refers to the maximum and minimum value of the variable^42,49^.

### Machine learning design

#### Supervised machine learning design

**Figure 3:**
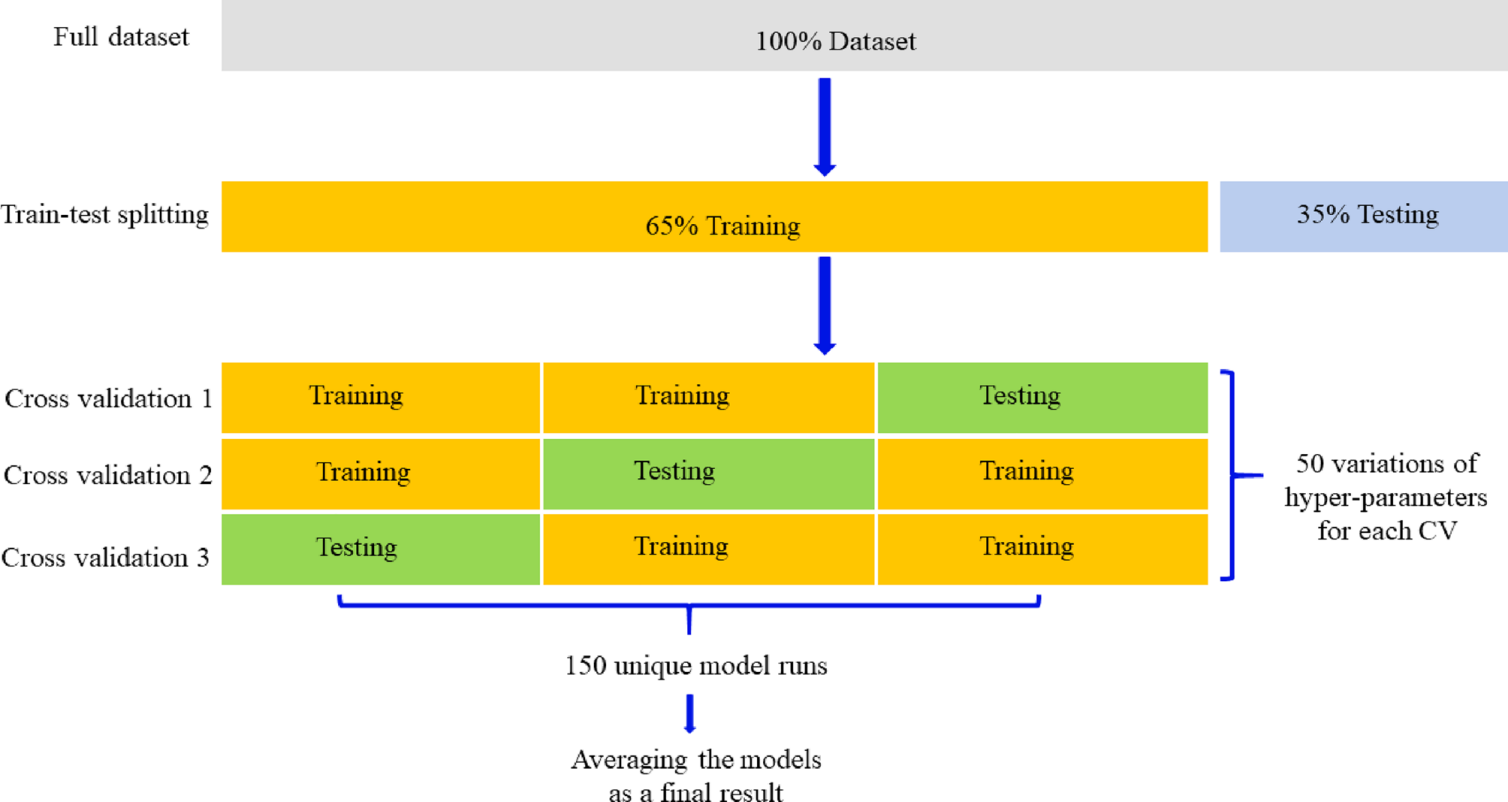
Threefold cross-validation scenario.

In the supervised machine learning section of this study, 212 datasets will be split into two groups: 65% for training purposes and 35% for testing. As an effort to prevent overfitting and leakage on the testing data both holdout and k-fold cross-validation method are adopted in this study to serves a dual purpose: ensuring an unbiased evaluation of the model and a thorough assessment of its generalizability. The holdout method provides a clean dataset for final model evaluation, free from any influence of the training process. Meanwhile, k-fold cross-validation is applied to the training data to reduce the potential variance in model performance that could result from a single train-test split, particularly important in datasets of limited size. This nested approach is a robust strategy for hyperparameter tuning, enabling the model to demonstrate consistent performance across multiple subsets of the data, thus reinforcing its ability to generalize beyond the training sample. In this scenario, the model will continue to be trained until all folds have been used for testing once. The average score of the testing fold will recognize as validation score (Fig. 3)^50^.

The algorithm's hyperparameters will also be tuned to determine the optimal model for each method^18^. The set of each individual's input hyper-parameters is displayed in Tables 2, 3, 4 and 5.

**Table 2:** Hyper-parameter tuning for random forest (RF).

Hyper parameter	Code/symbol	Minimum	Maximum	Best value
Maximum tree	algo__n_estimators	100	200	151
Maximum depth	algo__max_depth	20	80	70
Maximum features	algo__max_features	0.1	1	0.12828
Minimum sample leaf	algo__min_samples_leaf	1	20	7

**Table 3:** Hyper-parameter tuning for extreme gradient boosting (XGB).

Hyper parameter	Code/symbol	Minimum	Maximum	Best value
Maximum tree	algo__n_estimators	100	200	185
Maximum depth	algo__max_depth	1	20	3
Learning rate	algo__learning_rate	− 2	0	0.12164
Maximum features	algo__max_features	0.1	1	0.7865
Gamma	algo__gamma	1	10	1
Alpha	algo__reg_alpha	− 3	1	0.0115
Lambda	algo__reg_lambda	− 3	1	0.0094

**Table 4:** Hyper-parameter tuning for SVM (support vector machine).

Hyper parameter	Code/symbol	Minimum	Maximum	Best value
Gamma	algo__gamma	− 3	3	0.4374
Regularization parameter	algo__C	− 3	3	12.746

**Table 5:** Hyper-parameter tuning ANN (artificial neural network).

Hyper parameter	Code/symbol	Minimum	Maximum	Best values
Hidden layer size	mlp__hidden_layer_sizes	(16, 8, 4)	(8, 4)	(16, 8, 4)
Learning rate	mlp__learning_rate_init	0.001	0.01	0.01
Alpha	mlp__alpha	0.0001	0.003	0.0003

#### Unsupervised machine learning design

In the unsupervised machine learning section, the distribution of the log FZI data will be examined through a histogram and a normal probability plot to make initial judgments regarding data clustering. A statistical method incorporating the normal probability plot will be employed, where a straight line in the plot signifies a normal distribution. If multiple straight lines with varying slopes are present, it indicates the existence of different datasets that share the same normal distribution, implying the presence of distinct clusters.

To determine the optimal number of clusters in the K-Means algorithm, the elbow criterion is utilized. The elbow criterion suggests selecting the number of clusters where the addition of another cluster does not significantly contribute new information^51^. In this study, the elbow method incorporates the Root Mean Square Error (RMSE) and R-squared as measures to evaluate the clustering of flow units^52^. These metrics quantify the deviation between observed and estimated values, providing insights into the optimum cluster numbers for reservoir characterization of hydraulic flow units. Several previous studies have utilized the elbow method in conjunction with the RMSE and R-squared metrics to determine the optimal number of clusters for hydraulic flow units in reservoir characterization efforts^6,53,54^.

**Figure 4:**
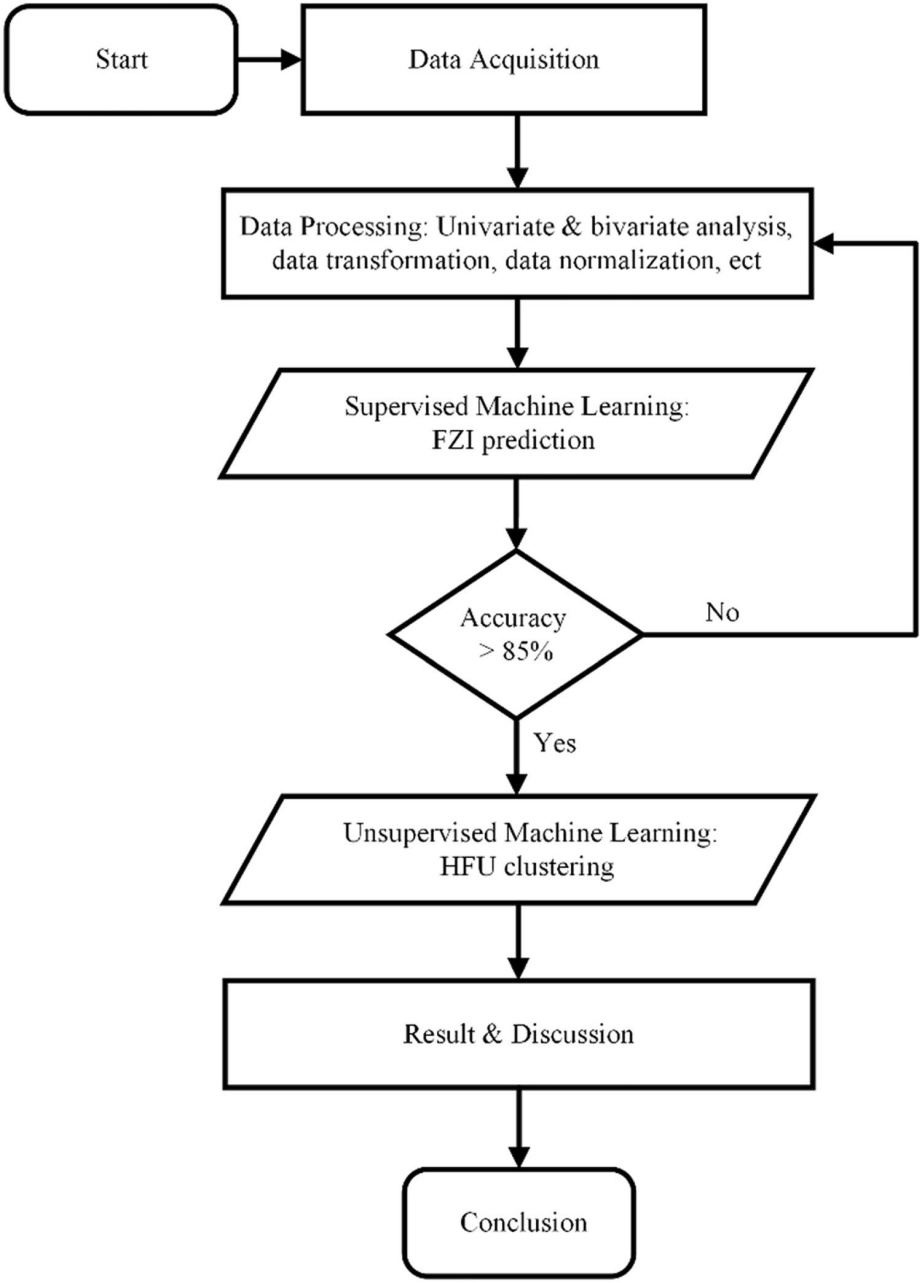
Workflow of the study for supervised and unsupervised machine learning models.

It is crucial to follow an organized process to obtain precise and trustworthy outcomes. Figure 4 displays the precise study methodology in detail.

## Result and discussion

### Supervised machine learning for flow zone indicator prediction

#### Pre-processing machine learning model

In this work, a predictive model is constructed using three-fold cross-validation and hyperparameter optimization. Randomized search cross-validation is used as a solution to Grid Search Cross-Validation (Exhausted Cross Validation) to reduce computation time when performing hyperparameters^55^. Using this approach, 50 iterations of hyper-parameters are paired with 3 folds of cross-validation to generate 150 training models, which are then assessed using coefficient determination metric evaluation (R^2^).

**Figure 5:**
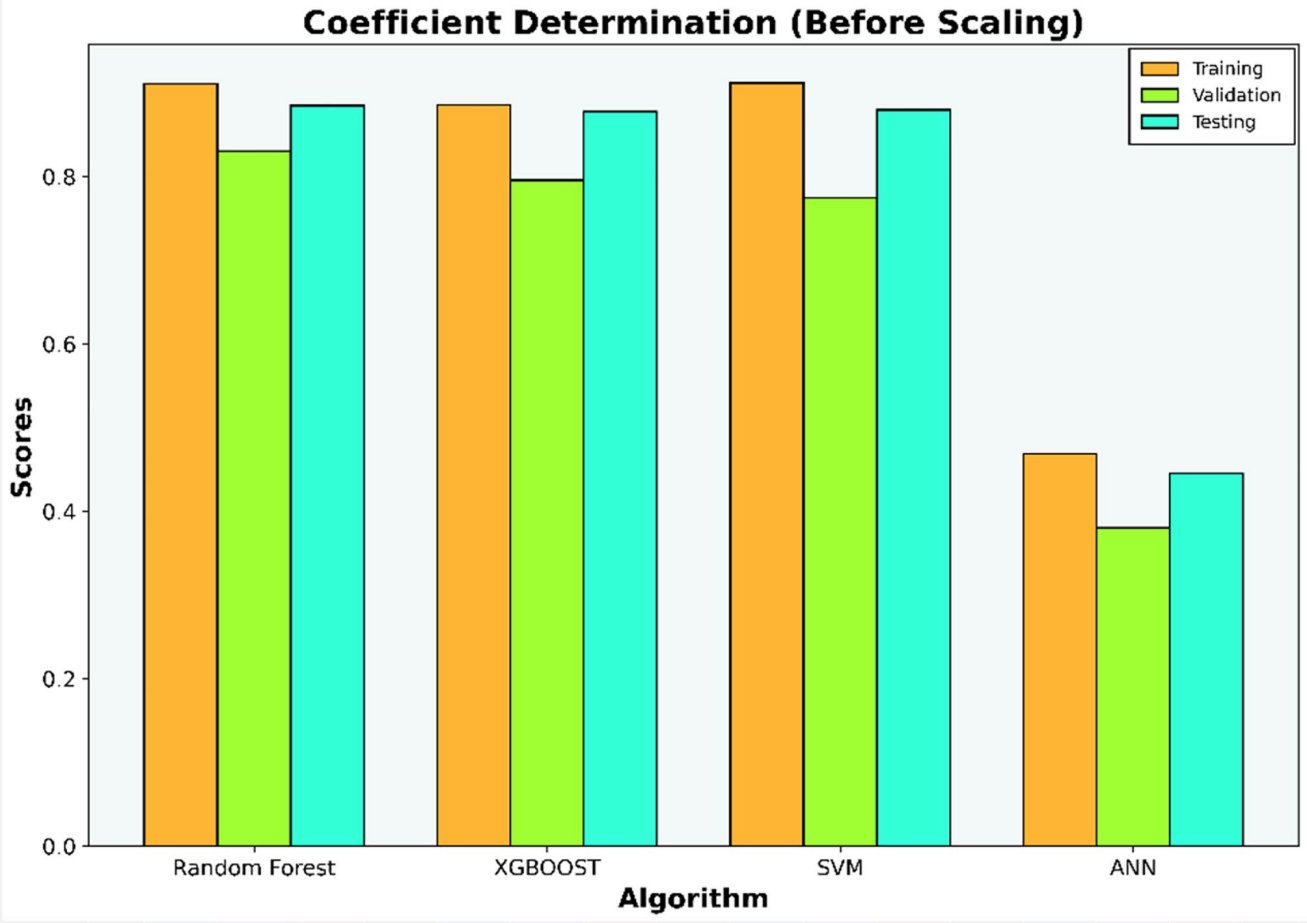
Coefficient determination (R^2^) summary for the different ML methods using unscaled datasets.

**Figure 6:**
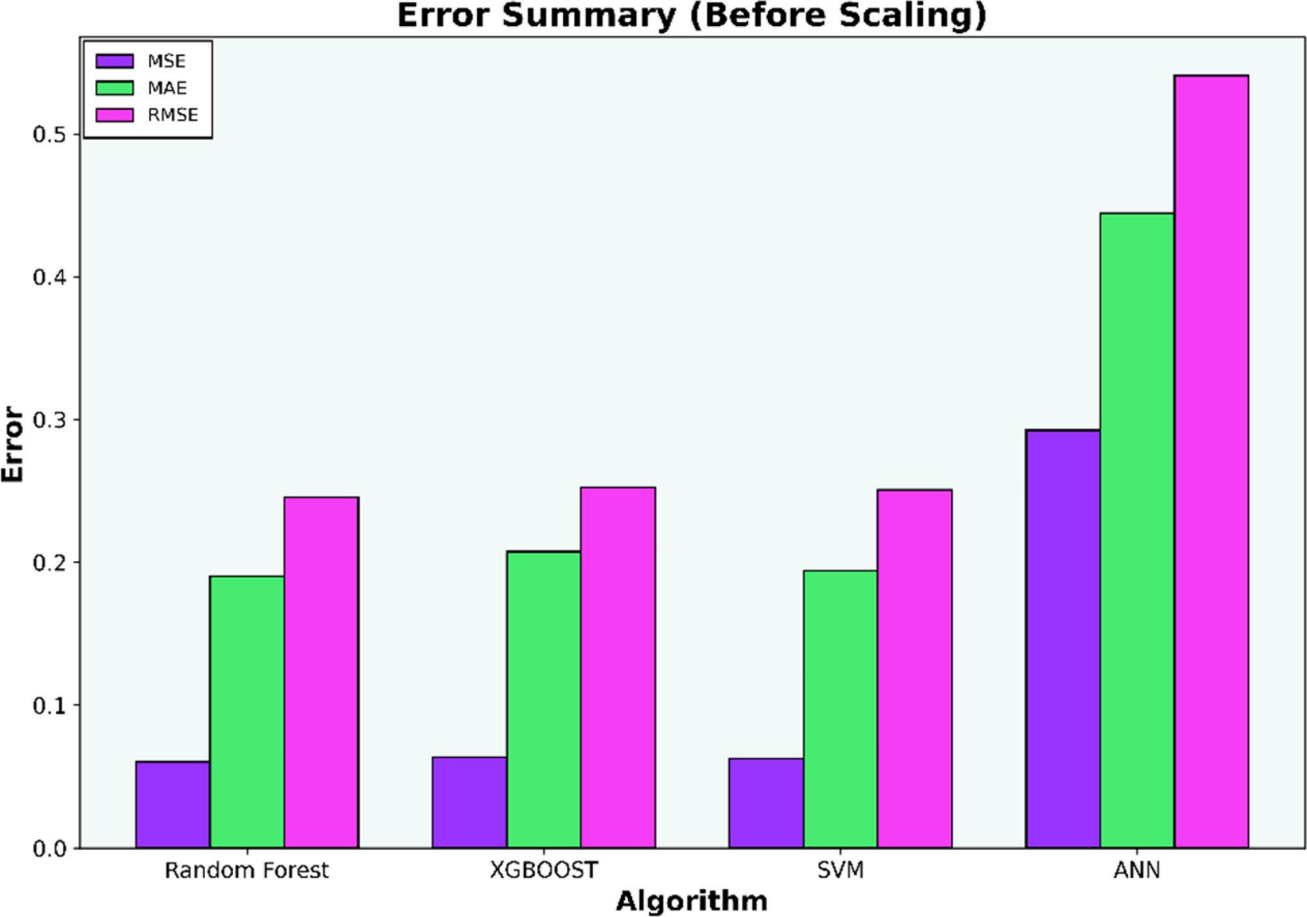
Error summary for the different ML methods using unscaled datasets.

**Figure 7:**
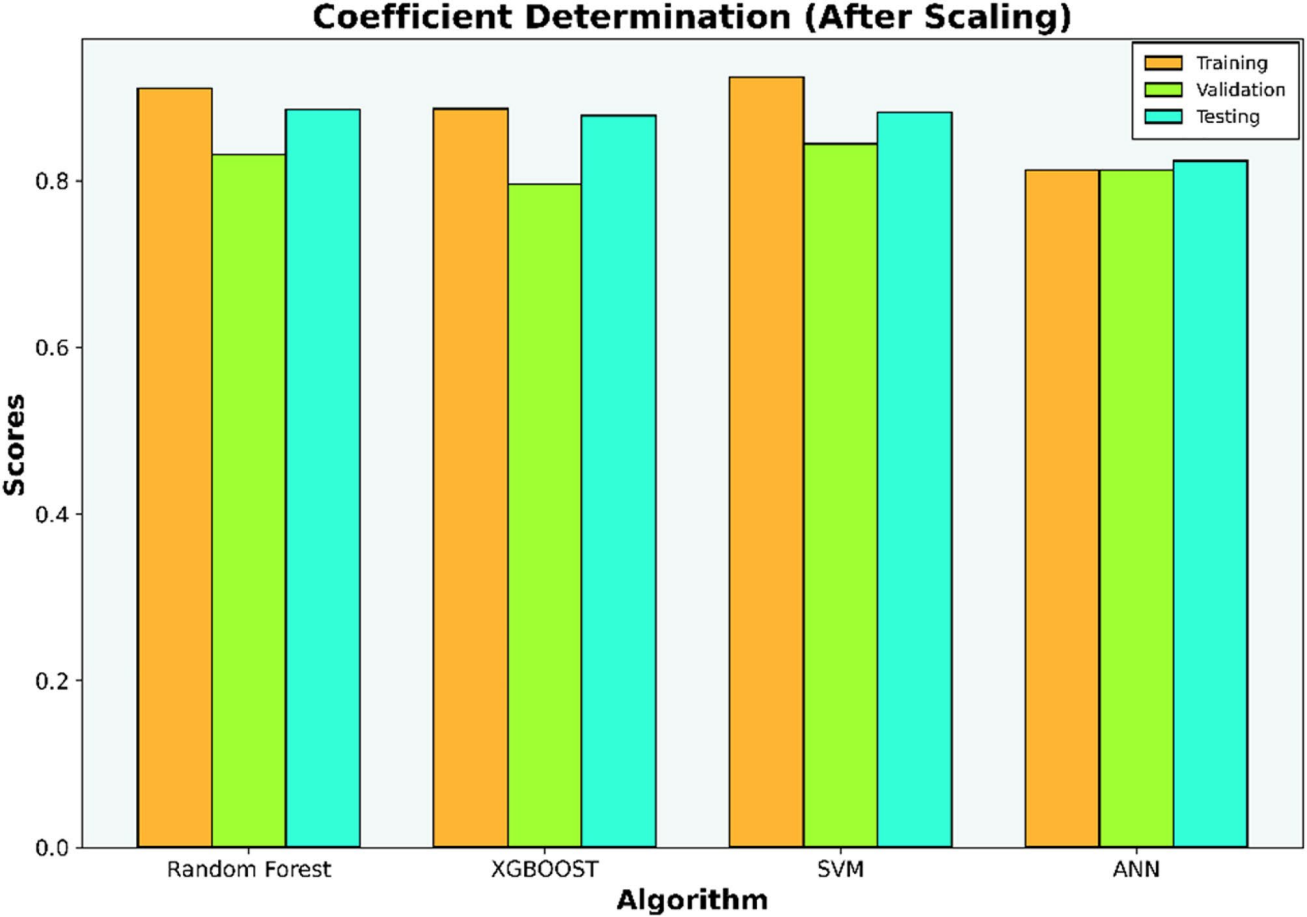
Coefficient determination (R^2^) summary for the different ML methods using scaled datasets.

**Figure 8:**
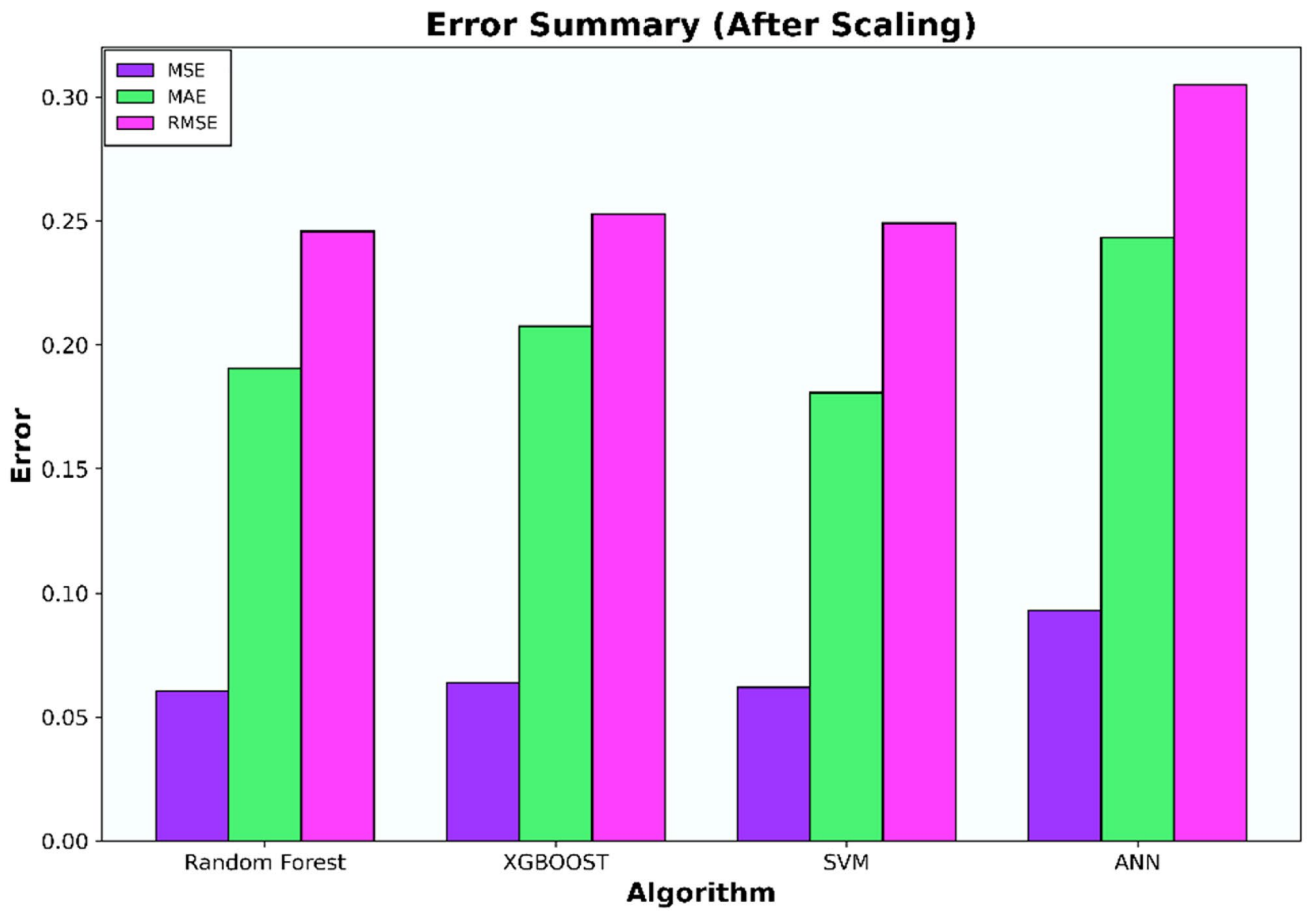
Error summary for the different ML methods using scaled datasets.

The initial investigation will compare models that have undergone scaling to those that have not. Evaluation metrics such as R-squared (R^2^), Mean Squared Error (MSE), Mean Absolute Error (MAE), and Root Mean Squared Error (RMSE) for each algorithm are compiled in Tables 6, 7, 8, and 9 and illustrated in Figs. 5, 6, 7, and 8.

**Table 6:** Coefficient determination (R^2^) summary (before scaling).

Model	Training	Validation	Testing
Random forest	0.9106	0.8310	0.8850
XGB	0.886	0.796	0.8780
SVM	0.912	0.775	0.8800
ANN	0.469	0.3805	0.4458

**Table 7:** Error summary (before scaling).

Model	MSE	MAE	RMSE
Random forest	0.06	0.19	0.25
XGB	0.06	0.21	0.25
SVM	0.06	0.19	0.25
ANN	0.29	0.44	0.54

**Table 8:** Coefficient determination (R^2^) summary (before scaling).

Model	Training	Validation	Testing
Random forest	0.91	0.83	0.89
XGB	0.89	0.80	0.88
SVM	0.92	0.84	0.88
ANN	0.81	0.78	0.82

**Table 9:** Error summary (after scaling).

Model	MSE	MAE	RMSE
Random forest	0.06	0.19	0.25
XGB	0.06	0.21	0.25
SVM	0.06	0.18	0.25
ANN	0.09	0.24	0.31

The outcomes presented in Figs. 5 and 7 indicate that applying scaling techniques improves the performance of both the SVM and ANN models, with notable enhancement observed in the ANN. The SVM is recognized for its robustness, employing a margin-based loss function that effectively manages the dimensionality of the input space. However, SVM may underperform with skewed datasets as finding the optimal separating hyperplane becomes challenging with imbalanced data^56^. A similar challenge is observed with neural network algorithms, which, at their core, rely on linear regression principles. Extreme skewness in the data can substantially impact the performance of neural networks. Meanwhile the stability of the scores for both the Random Forest and XGBoost models, even after dataset standardization, can be attributed to their foundational decision tree structure. These models utilize bootstrapping sampling methods and an aggregation technique known as bagging to produce the final score. This approach equips the models with resilience against imbalanced or skewed datasets, ensuring consistent performance irrespective of data standardization^19–22^.

#### Data processing and features reduction

**Figure 9:**
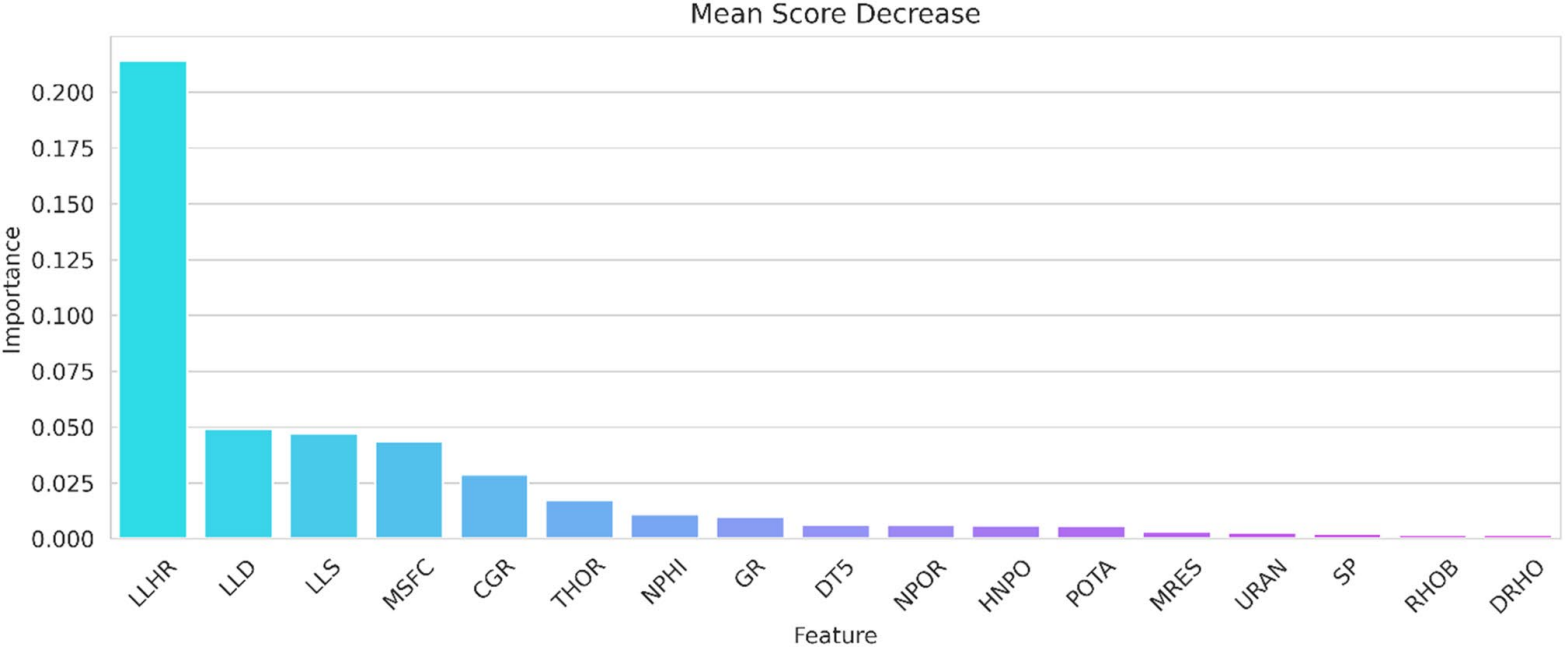
Feature importance analysis quantifying the significance of each parameter in the model construction.

To enhance machine learning model accuracy, various data processing techniques are used. Parameter reduction is achieved by analyzing the impact of excluding each variable using the feature importance method. The Feature Importance Analysis is performed using the random forest model as the benchmark to identify the most important parameters in the dataset. The random forest model is selected for its high accuracy, as indicated by the high R-squared values observed during the pre-processing stage of the data in both the training and testing sets. Figure 9 presents the results of the analysis, showing the relative importance of each input parameter in the dataset.

Figure 9 displays the relative importance of each parameter in the output model, as determined by a feature importance analysis using a random forest model. This analysis calculates the decrease in the Mean Squared Error (MSE) of the prediction, where a higher importance score indicates a greater role of the parameter in reducing the MSE^19–22^. LLHR emerges as a notable parameter with an importance score of 27%. It is important to understand that this score does not imply that excluding LLHR would directly result in a 27% change in the model's performance. Instead, it signifies LLHR's relative contribution to enhancing the model's predictive accuracy by reducing the MSE. The parameter selection process in this study was guided by the aim to include input parameters that collectively have a substantial impact on the model's effectiveness. The cumulative relative importance from LLHS to HNPO is 51%, indicating their combined significance in the model. Therefore, the final set of selected input parameters, comprising LLHR, LLS, MSFC, LLD, CGR, NPHI, THOR, NPOR, and HNPO, was chosen based on their collective ability to decrease the MSE and improve the model's overall predictive performance, rather than solely on their individual importance scores.

The feature importance analysis results are in line with the existing literature, as these parameters demonstrate a strong relationship with the calculation of FZI using a physics-based approach. The LLHR (Laterolog High-Resistivity), LLS (Laterolog Shallow), and LLD (Laterolog Deep) logs are crucial resistivity logs utilized in formation evaluation^57^ explored the relationship between resistivity and permeability using known water saturation and the apparent formation factor. The results of the study demonstrated a strong relationship between resistivity and permeability. The MSFC log provides quantitative resistivity data at a micro-scale and can be converted into visual images, allowing for detailed core permeability description through visual examination. Bourke^58^ observed a strong visual correlation between micro resistivity and permeability images, indicating their potential for capturing porosity–permeability variations. Micro resistivity data offer high-resolution permeability transformation, surpassing traditional logs, and have been used in various studies for permeability assessment and characterization. These findings highlight the significance of the MSFC log in permeability prediction.

Yao and Holditch^59^ established a correlation between core permeability and open-hole well-log data, highlighting the significance of the relationship between gamma-ray and permeability estimation, which ultimately contributes to the estimation of FZI. Thus, CGR is an important parameter in this model. NPHI, NPOR, and HNPO are different versions of thermal neutron porosity logs widely used for characterizing reservoir porosity. These logs have been extensively studied in combination with other parameters to determine lithology and estimate clay volume that reflects its importance for FZI prediction. The THOR (Thorium Concentration) log measures the thorium concentration in parts per million (ppm) using energy emissions from radioactive minerals, which are detected by the spectral gamma ray log. According to^60^, high concentrations of thorium are indicative of dark, organic-rich shale, as well as calcareous, brittle, and fractured shale. Hence, Thorium concentrations directly influence permeability and porosity and the rock type.

In addition to parameter reduction, data transformation using the Yeo-Johnson method was applied to the dataset. This transformation technique is employed to address the issue of non-normality in the data distribution. By employing this transformation, the data distribution becomes more symmetrical, thus meeting the assumptions of certain statistical models and improving the accuracy of subsequent analyses.

#### Post-data-processing machine learning model

**Figure 10:**
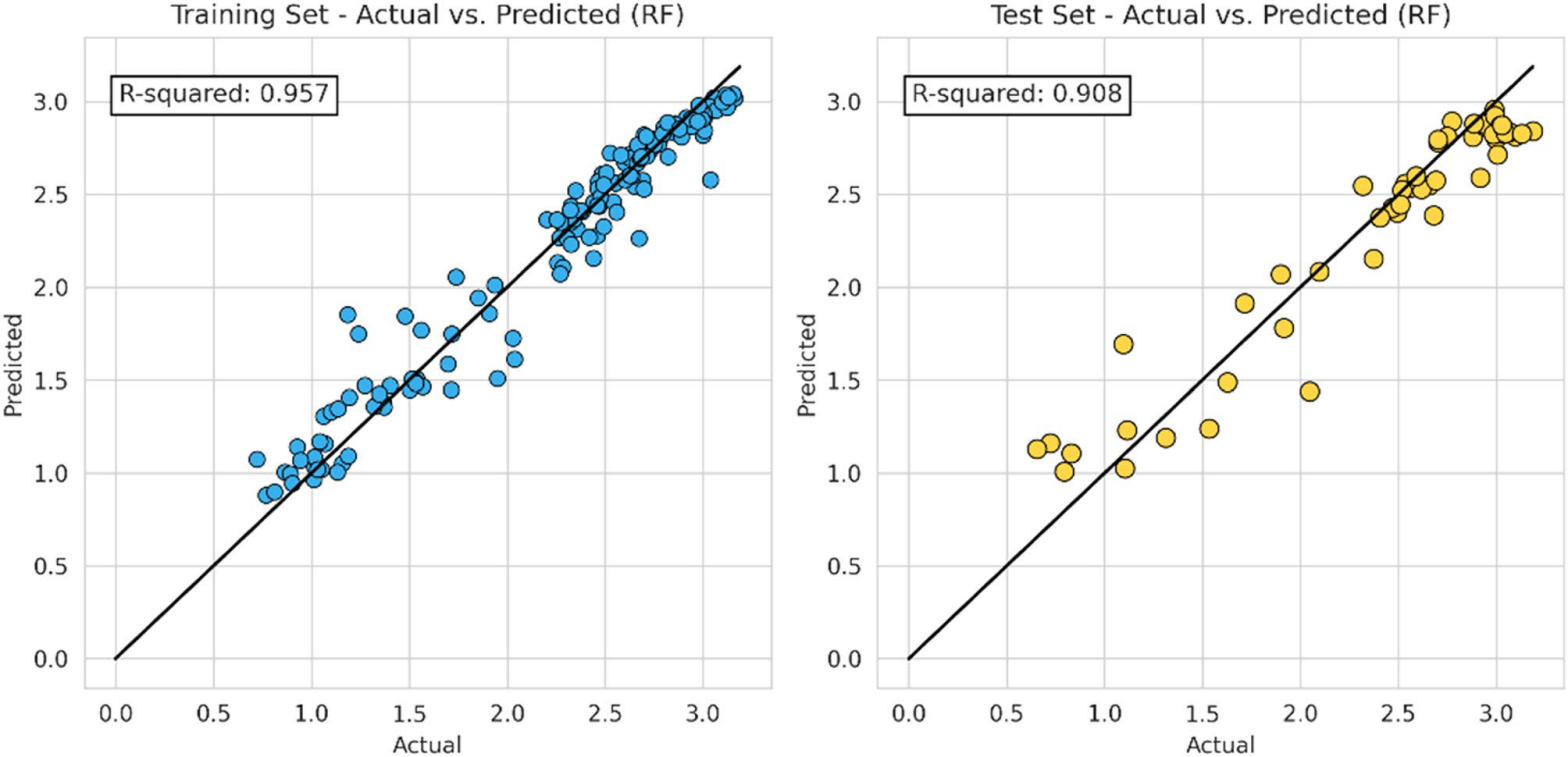
Coefficient determination (R^2^) random forest model.

**Figure 11:**
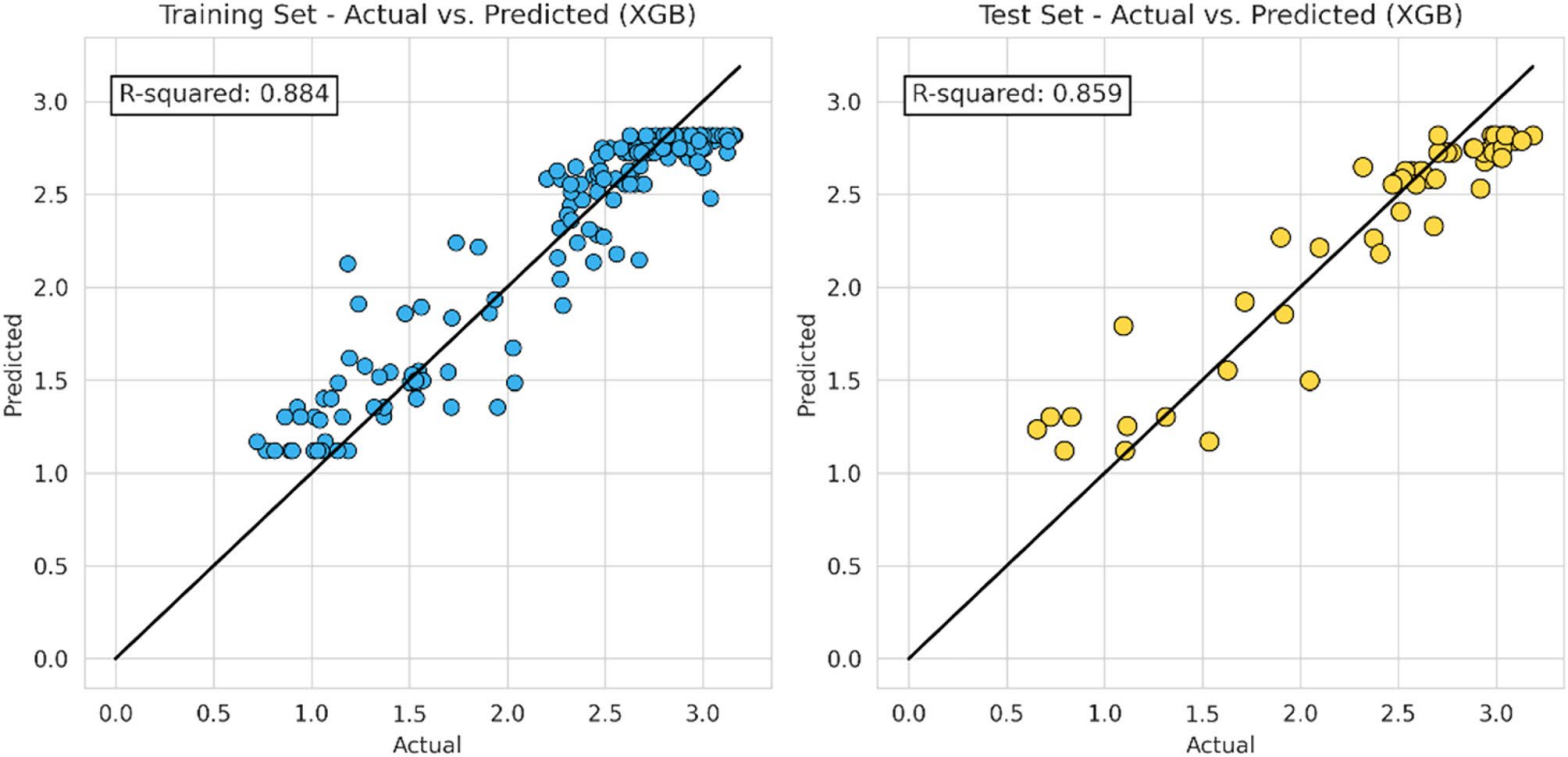
Coefficient determination (R^2^) XGB model.

**Figure 12:**
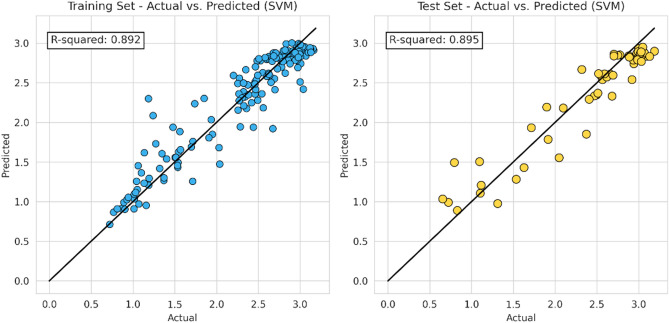
Coefficient determination (R^2^) SVM model.

**Figure 13:**
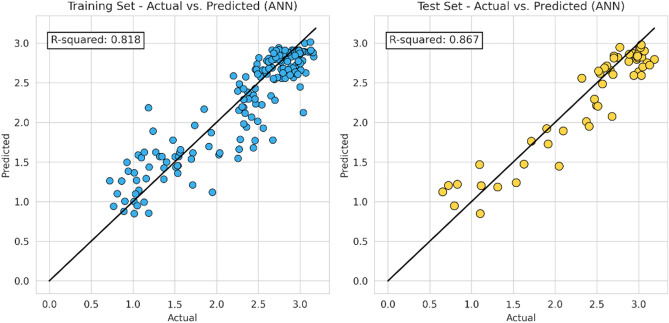
Coefficient determination (R^2^) ANN model.

**Figure 14:**
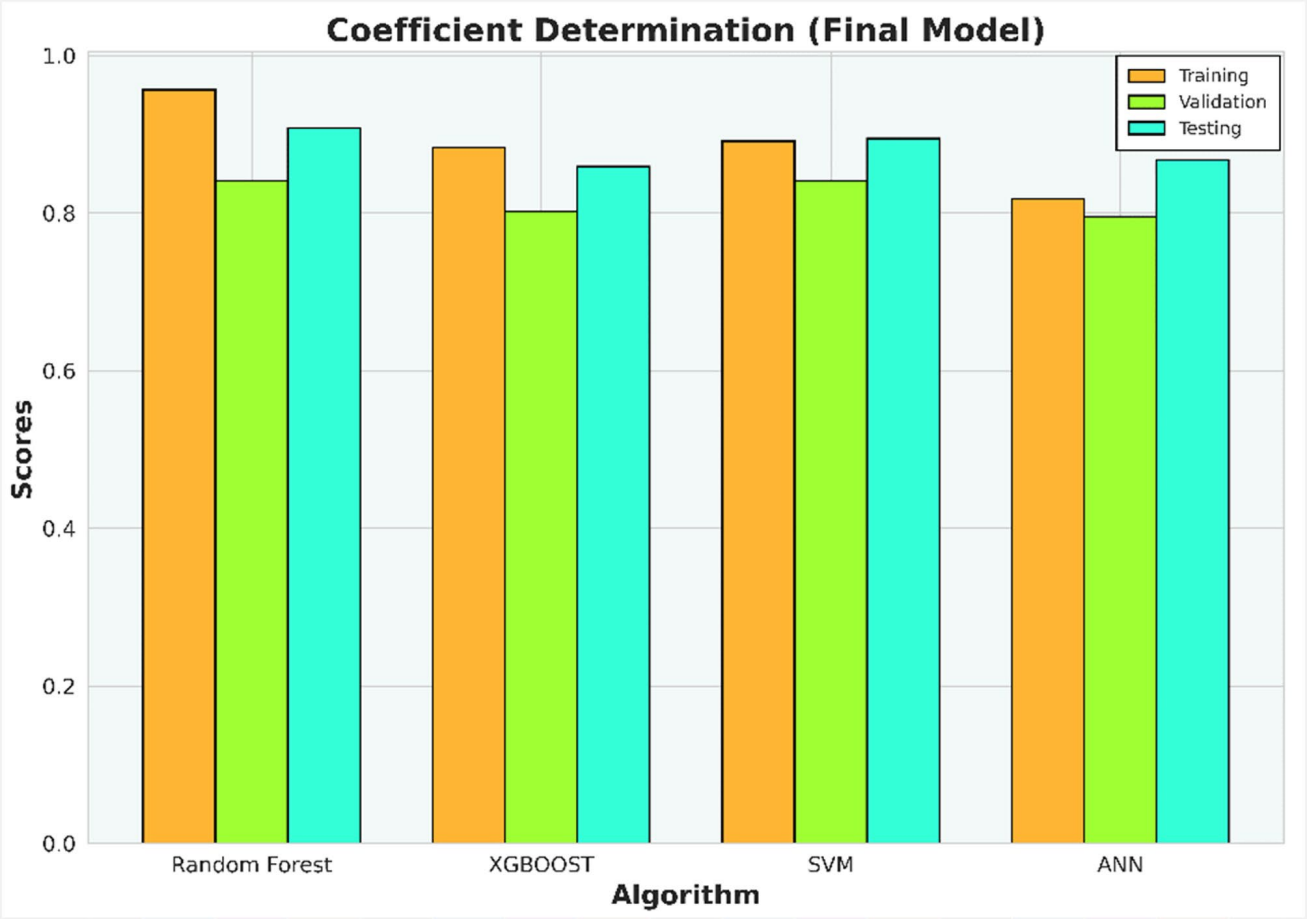
Coefficient determination (R^2^) summary (final model).

**Figure 15:**
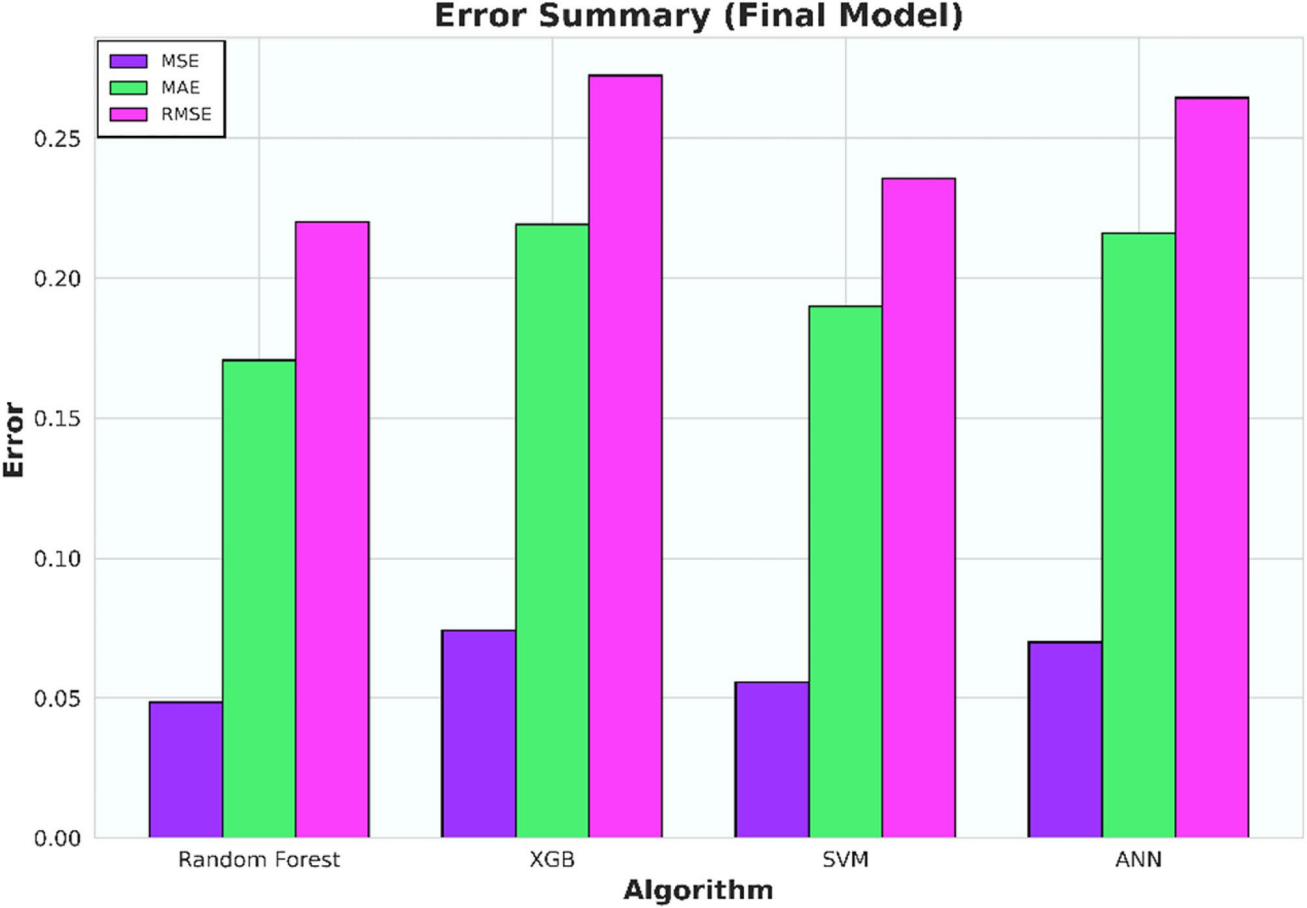
Error summary (final model).

In this step, the machine learning model proceeds to apply the same characteristic (hyper-parameter combination) as the previous model. The following Figs. 10, 11, 12 and 13 represent the results of the machine learning algorithm that was applied following data processing. These cross-plot figures showed the capabilities of the different machine learning to predict the flow zone index, where most of the data are aligned with the 45-degree line. Additionally, Figs. 14 and 15 and Tables 10 and 11 summarize the comparison models' performance post scaling and transformation process.

**Table 10:** Coefficient determination (R^2^) summary (final model).

Model	Training	Validation	Testing
RF	0.96	0.84	0.91
XGB	0.88	0.80	0.86
SVM	0.89	0.84	0.89
ANN	0.82	0.80	0.87

**Table 11:** Error summary (final model).

Model	MSE	MAE	RMSE
Random forest	0.05	0.17	0.22
XGB	0.07	0.22	0.27
SVM	0.06	0.19	0.24
ANN	0.07	0.22	0.26

Model evaluations demonstrate steady efficacy throughout the training, validation, and testing stages. The Random Forest model stands out with the highest accuracy in training and testing, at 0.9566 and 0.9081, respectively. Table 11 and Fig. 14 collectively suggest that the models retain high accuracy post-data processing. In Table 12 it can be seen the comparison of the final model to the initial model which did not undergo data processing, the final model that incorporated scaling and transformation exhibited enhancements. This is particularly noticeable in the case of the ANN model, which, as previously discussed, showed significant improvement. Due to the highest model performances resulting from the final model, it is recommended to use the post-processed models for future research, as they offer a well-tuned blend of dimensionality reduction and predictive capability.

**Table 12:** Relative differences of initial and final model score (R^2^).

Model	Training (%)	Validation (%)	Testing (%)
RF	5.05	1.22	2.61
XGB	− 0.27	− 2.13	0.72
SVM	2.25	8.45	1.66
ANN	74.43	109.15	94.57

### Unsupervised machine learning for hydraulic flow unit classification

#### Initial observation

**Figure 16:**
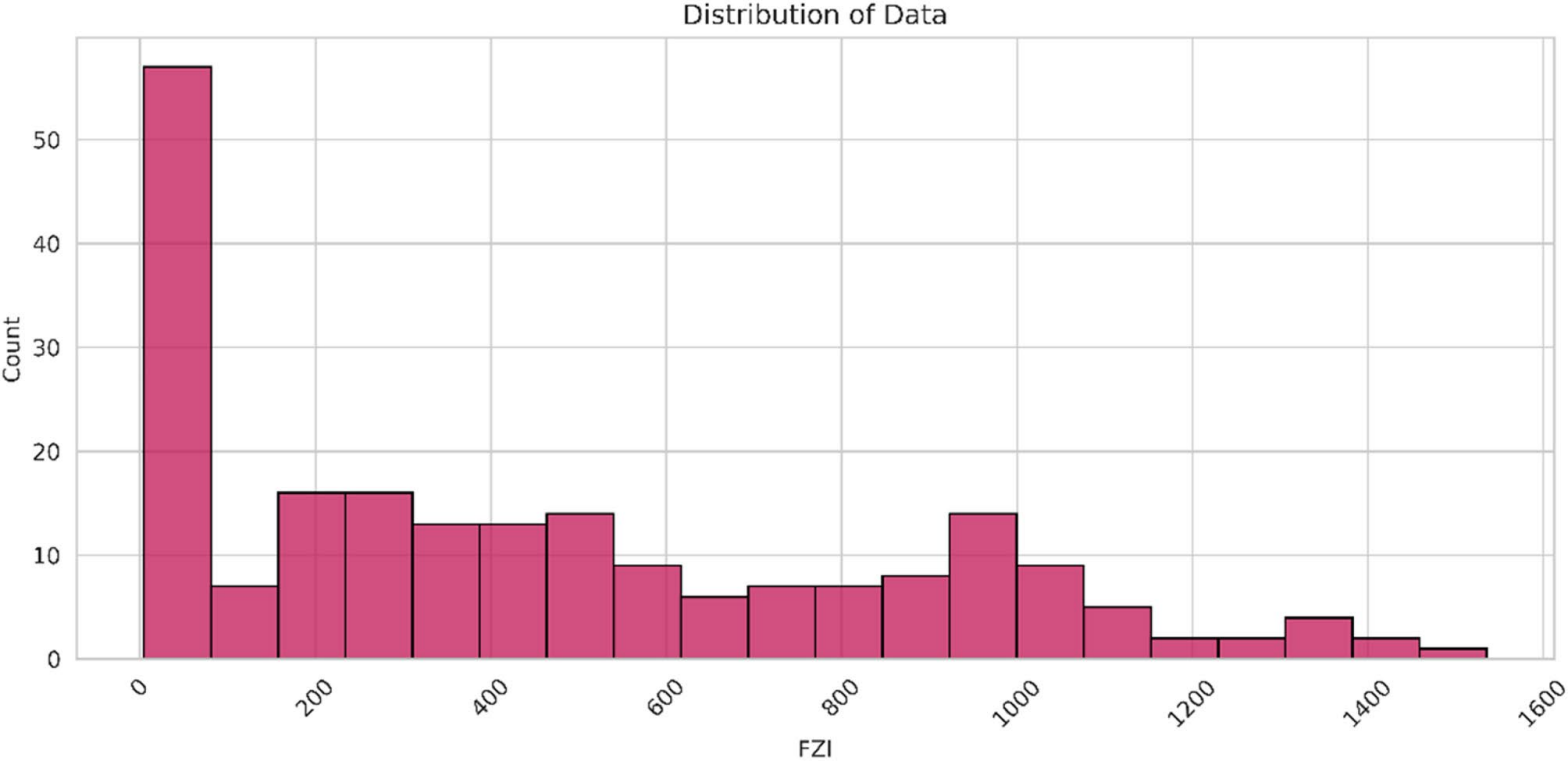
The distribution of FZI value.

**Figure 17:**
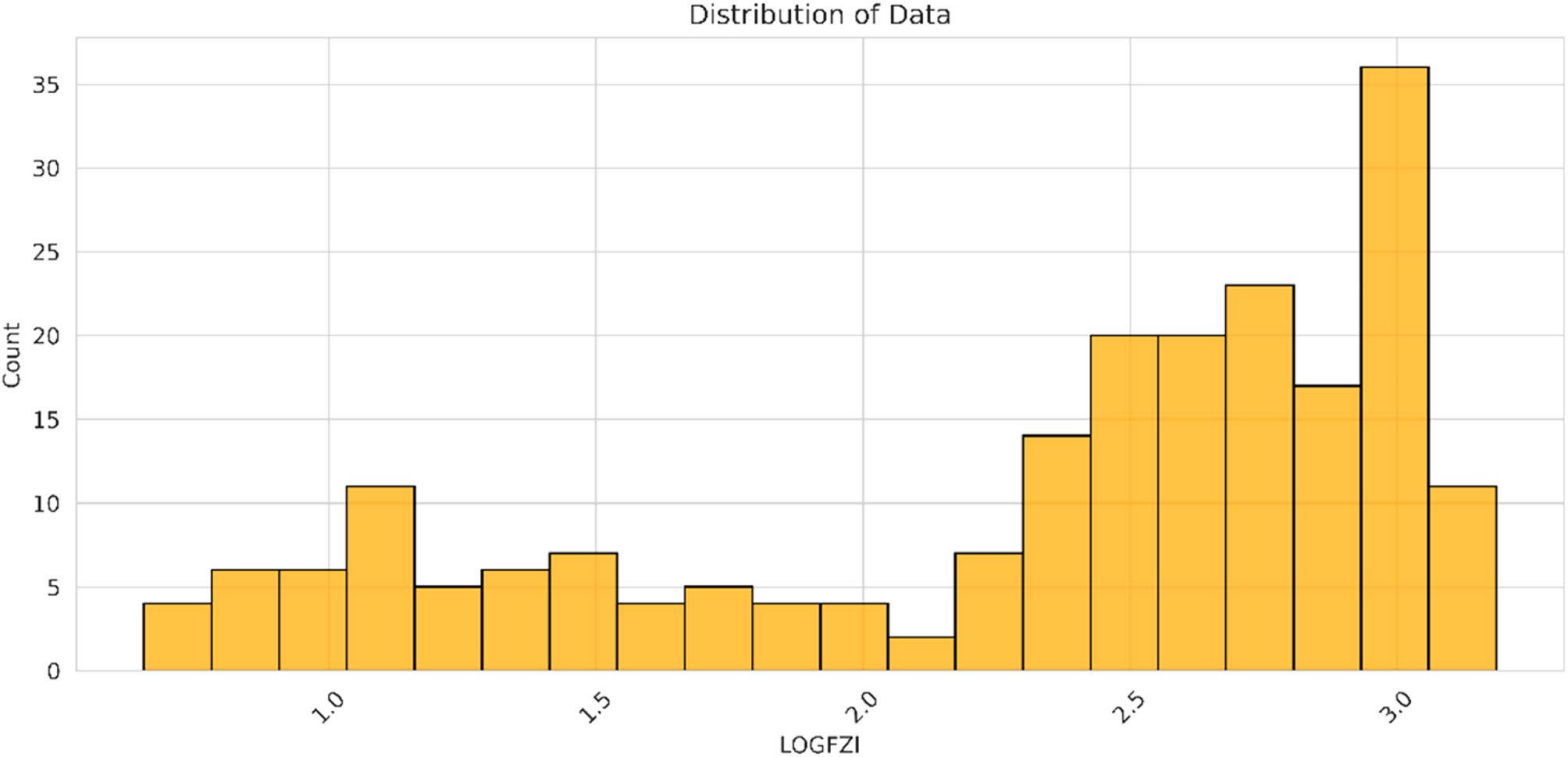
The histogram for log FZI data.

Figure 16 displays the histogram plot of FZI value, showing a non-normal distribution. Despite attempts to transform the heavily non-normal FZI data to log FZI (Fig. 17), the resulting distribution remains non-normal due to FZI being influenced by the direction of fluid flow (permeability) and requiring further averaging or upscaling methods. Consequently, determining the number of hydraulic flow units (HFUs) solely from this plot is challenging. The histogram represents overlapping individual normal distributions, necessitating the isolation and identification of these individual distributions to accurately estimate the number of HFUs^54^. Therefore, while the histogram provides insights into the variation of HFU distribution across the formation, it offers a qualitative analysis rather than a precise count of HFUs.

**Figure 18:**
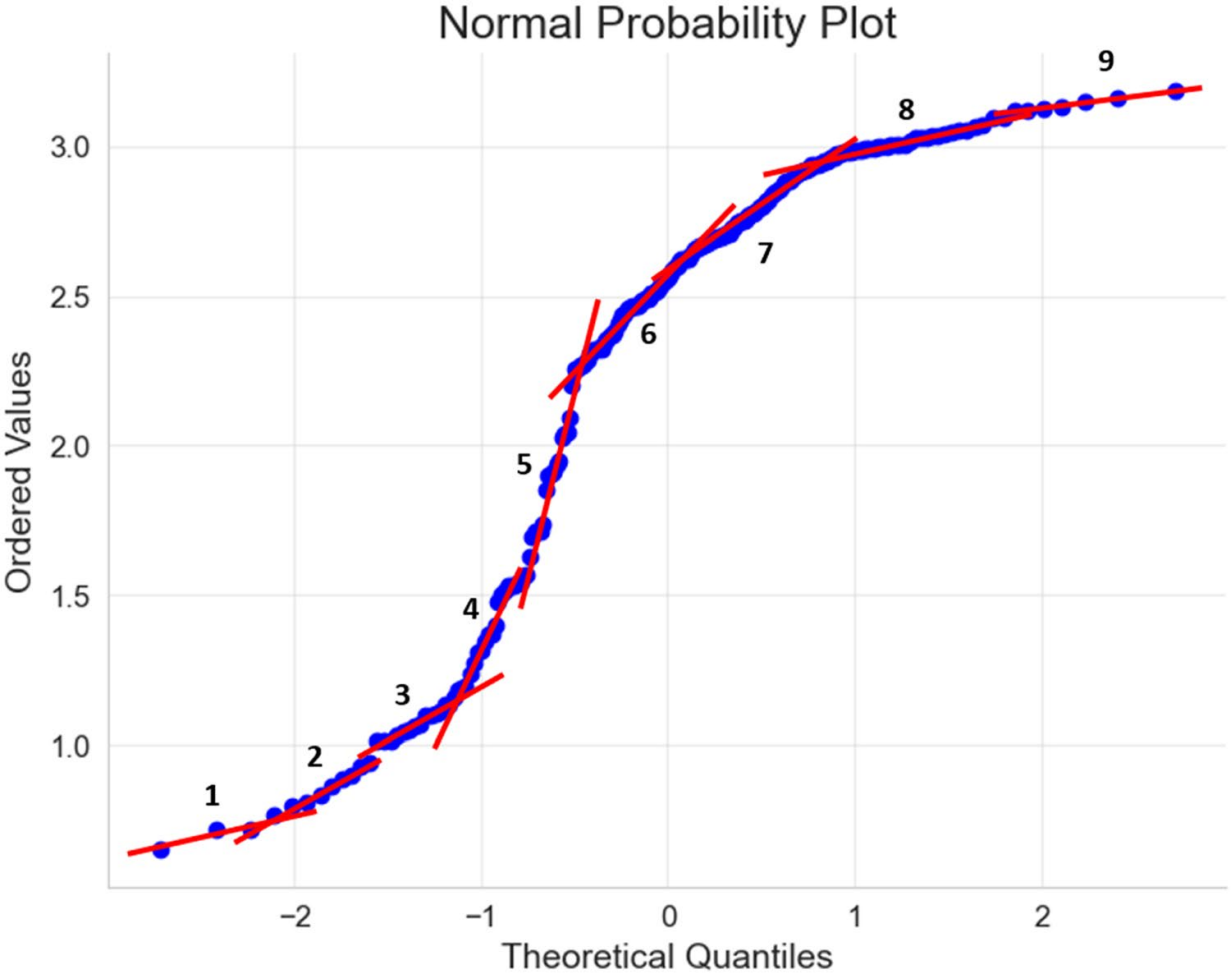
The normal probability plot for the log FZI data.

The normal probability plot is used as a statistical technique to assess the normality of a dataset. The presence of multiple straight-line segments in the plot indicates the presence of different hydraulic flow units (HFUs), each with its distinct normal distribution. Figure 18 displays nine distinct straight lines, suggesting the existence of nine HFUs in the formation. However, it's important to note that this approach relies on statistical analysis and visual interpretation, which can be subjective. Caution should be exercised when interpreting the results. Despite its limitations, this method is a valuable tool in data analytics and provides insights into the properties and behavior of a reservoir.

#### The optimum cluster number

**Figure 19:**
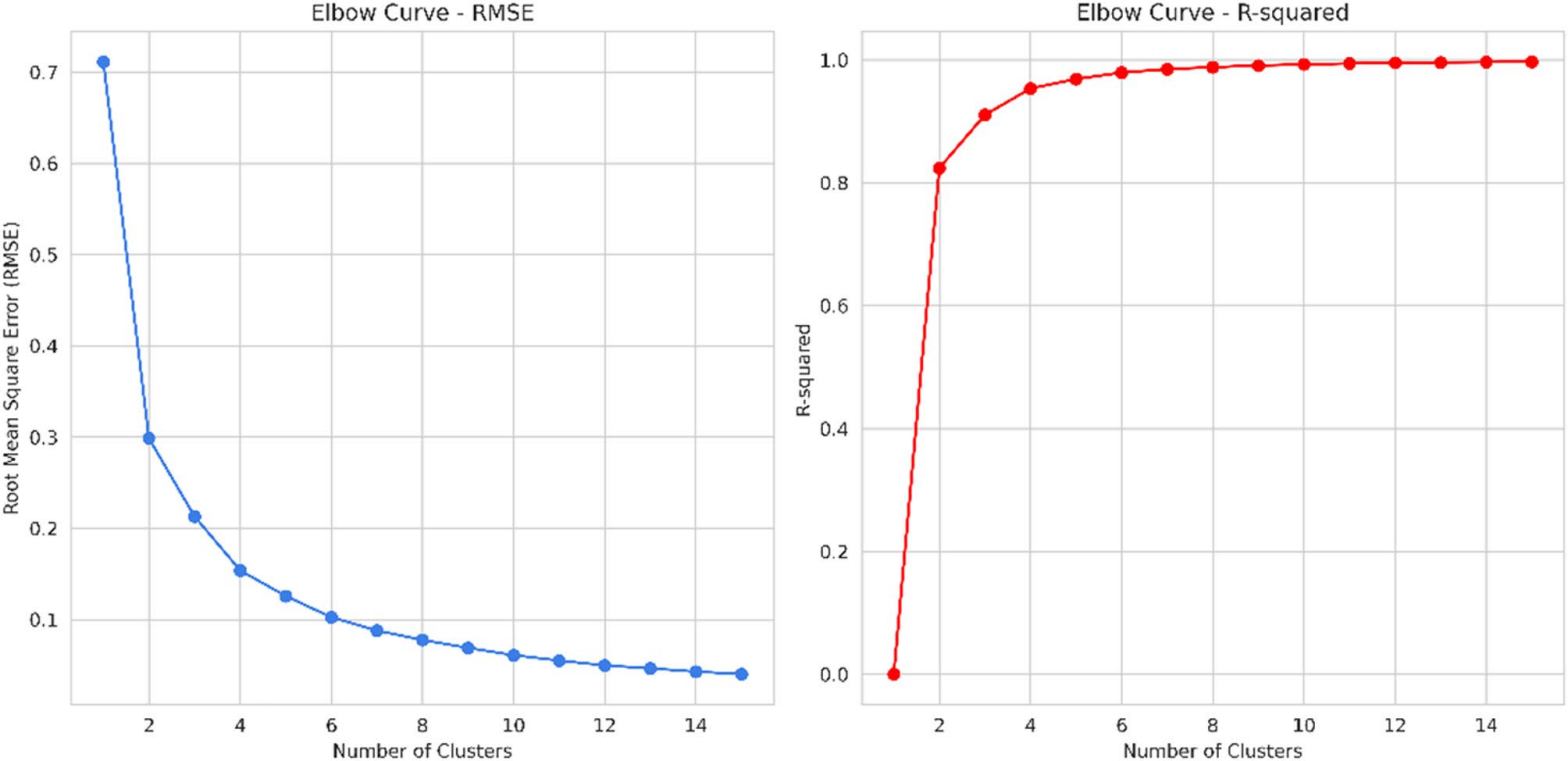
The Elbow method plot illustrates the RMSE and R-squared values.

In the initial stage of K-Means clustering, the elbow method is utilized to determine the optimal number of flow units (clusters). In this study, the elbow method uses RMSE and R-squared evaluations to determine the optimal number of flow units^61^. The results of the elbow method plot are displayed in Fig. 19.

Both the RMSE and R-squared approach may provide a different interpretation of the optimal HFU value. Considering the previously assessed heterogeneity, for the RMSE method, the optimum HFU value is taken as the number that has a minimum difference of 10% from the previous HFU value. Thus, the optimum HFU number for RMSE is 10, as, at 11, the value drops below 10%. In contrast, the R-squared method shows very small differences between R-squared values for each HFU number. Therefore, the interpretation of the R-squared method relies on visually observing the plot itself. By examining the plot, it is evident that an HFU of 10 exhibits the most horizontal straight line among all the previous ones. Consequently, the HFU value of 10 is considered the optimum value based on both the RMSE and R-squared approaches.

#### The K-means clustering

After determining the optimum cluster number, the K-Means clustering algorithm is utilized. The selected optimum HFU value is 10, and to ensure consistent and reproducible results, the random state parameter is set to 42 during the initialization of the K-Means clustering model. This parameter controls the random initialization of cluster centroids^62^. By using a specific random seed, the same initial centroids are used each time the code runs, ensuring consistent results and facilitating debugging and result comparison. Once the K-Means model is initialized with 10 clusters and the random state is set, the model is fitted to the data, allowing for further analysis and interpretation based on the resulting cluster assignments. The fitted data is then plotted in a log–log plot of RQI vs PHIZ, with each FZI value corresponding to its respective HFU unit.

**Figure 20:**
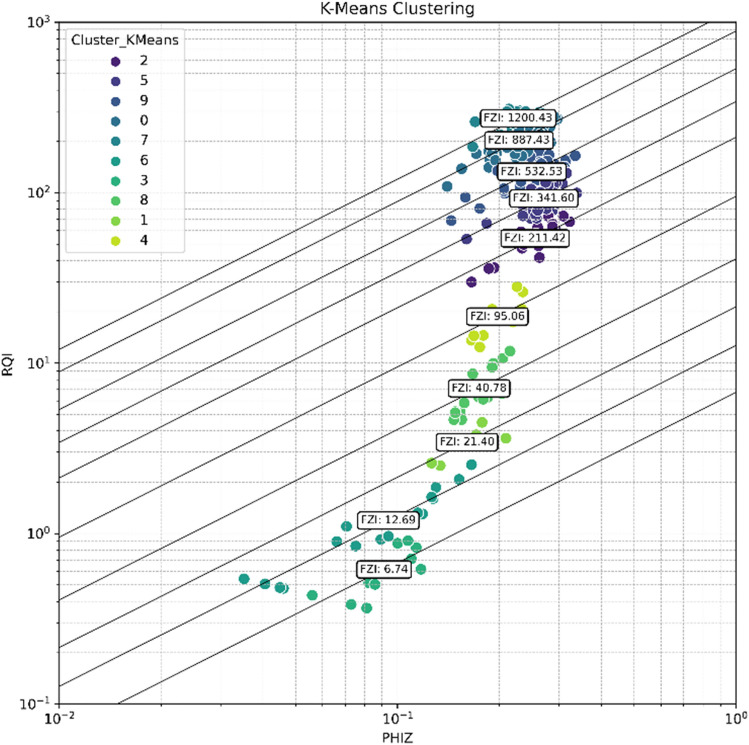
HFU clusterization using K-means method.

**Figure 21:**
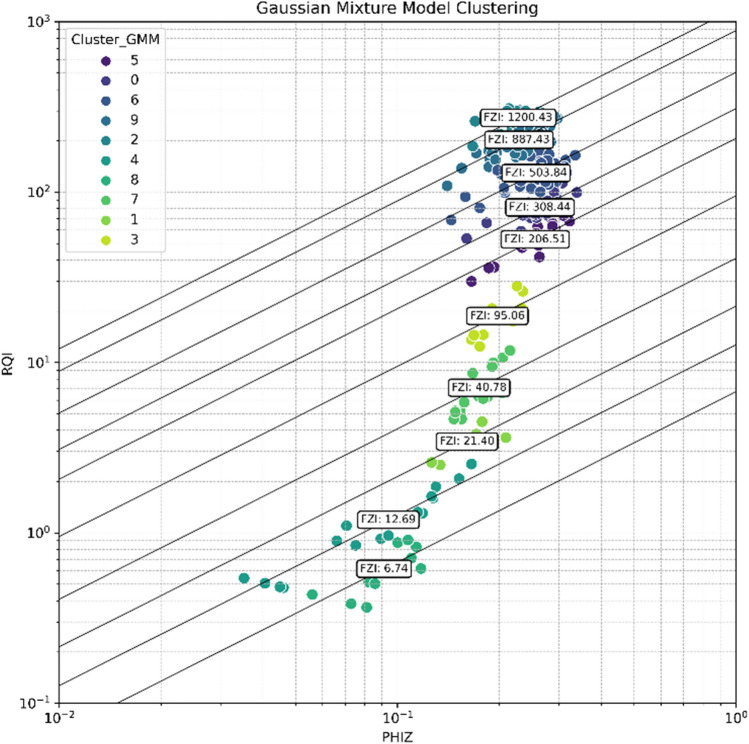
HFU clusterization using Gaussian mixture method.

Figures 20 and 21 display the results of dataset clustering using the K-means algorithm and the Gaussian Mixture Model (GMM), respectively. Upon examination of the figures, it is apparent that both algorithms have delineated similar clusters within the dataset. Although the labeling of the clusters differs between the two methods, the composition of the data points within corresponding clusters is largely analogous. This consistency between the K-means and GMM clustering outcomes suggests that both methods are capturing the inherent groupings within the dataset effectively. The parallelism in results reinforces the reliability of the clustering, affirming that the dataset possesses a structure that is robust to the clustering technique applied. The congruence of these clustering methods provides a validated foundation for further analysis.

**Figure 22:**
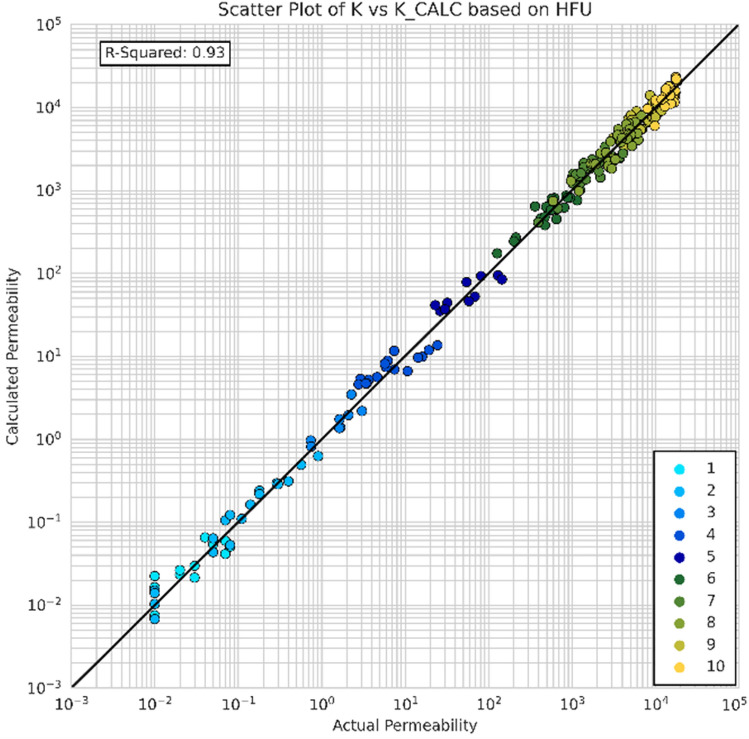
The predicted (calculated) vs actual permeability.

The performance of the evaluation and clustering method was assessed by calculating the permeability using the FZI values for each flow unit cluster by using Eq. (5). The calculated permeability was then compared to the actual permeability. Figure 22 displays the comparison between predicted and actual permeability values. The results indicate a high R-squared value of 0.93, demonstrating the effectiveness of the clustering method. This outcome validates the evaluation of reservoir heterogeneity, the determination of optimum HFU numbers, and the utilization of FZI for clustering. Table 13 provides the average permeability and porosity values for each flow unit cluster. It is important to note that when addressing heterogeneity, the choice of averaging method (arithmetic, harmonic, geometric) for permeability depends on the distribution of permeabilities within the rock during deposition^63^. By examining the FZI values alongside their respective average permeabilities, it is possible to predict the permeability quality of a specific location, thus enabling an assessment of its potential for fluid flow.

**Table 13:** Average porosity and permeability for each HFU.

HFU	Number of samples	FZI	Mean porosity	Permeability		
				Arithmetic	Harmonic	Geometric
1	12	7	0.08	0.035	0.024	0.03
2	18	13	0.08	0.176	0.036	0.092
3	8	21	0.14	1.7	1.4	1.5
4	16	41	0.15	7.7	6.9	7.3
5	10	95	0.16	60	52	56
6	21	211	0.20	624	502	568
7	41	341	0.20	1444	1960	762
8	35	532	0.19	3794	2806	3334
9	40	887	0.18	8184	6410	7314
10	21	1200	0.18	14,299	12,511	13,446

### Models validation and applications in unsampled formations

**Figure 23:**
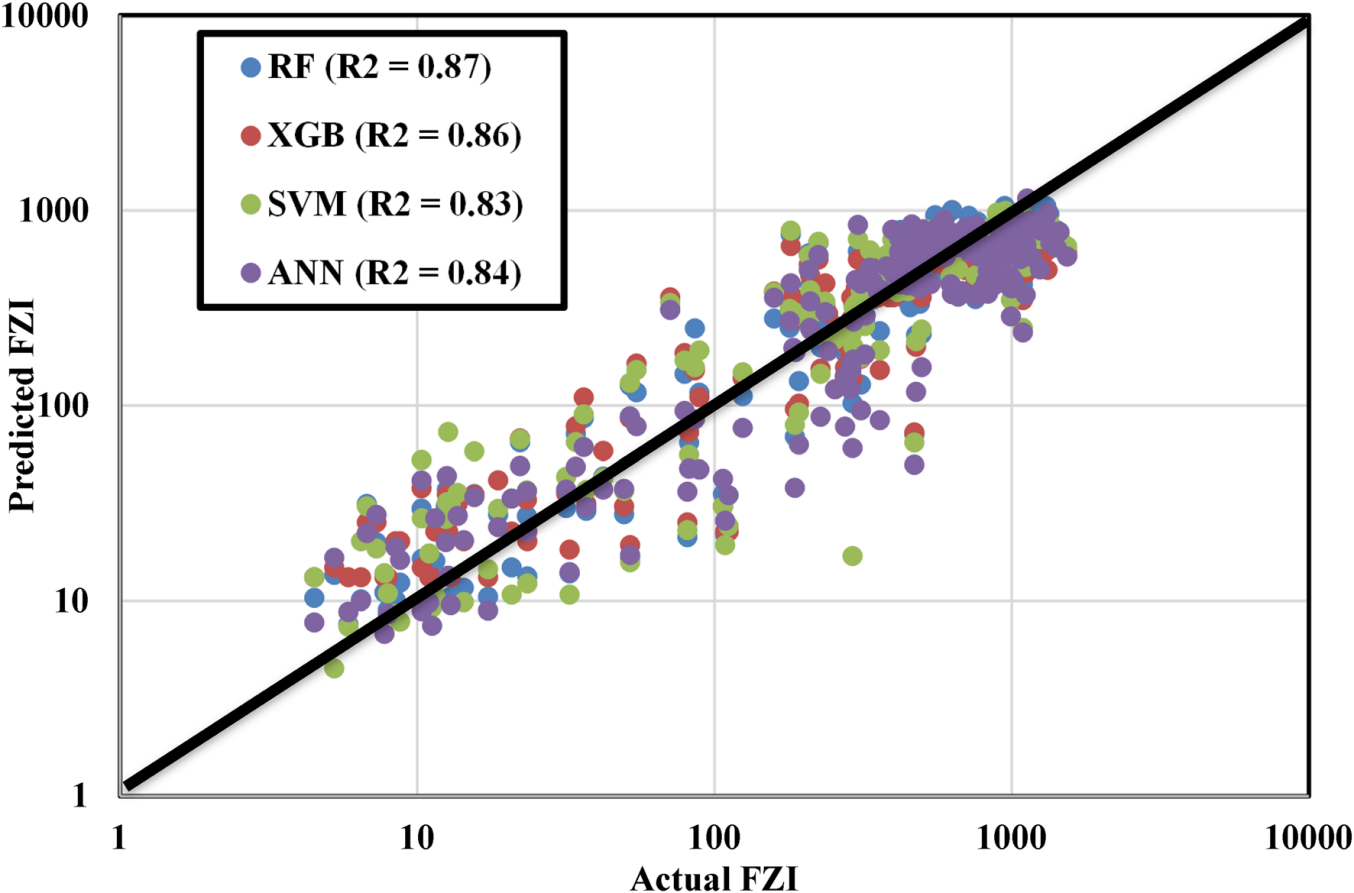
Different algorithm performance for additional unseen data.

Figure 23 shows the results of applying four models to the additional unseen location. The trend reveals that the ANN model performs the poorest, followed by the SVM, while the XGB and RF models exhibited the highest performance. This result is consistent with the values presented in Tables 10 and 11, which showed that the RF model is the optimal choice for predicting the FZI. This decision is based on the highest R-squared and lowest error score values obtained from both the training and testing datasets, and with their proximity indicating good generalization.

Several literatures further support this decision by acknowledging the acceptability of different models after data processing, as each model possesses strengths based on the nature of the data. The superior performance of the Random Forest model over others, particularly SVM and ANN, in this study is likely due to the characteristics of the dataset. Moreover, the dataset size used in this study is relatively small, comprising only 159 training and 53 testing data points. This condition is disadvantageous for algorithms like SVM, which depend on the spatial dimensions of the data, and for ANN models, which are based on fundamental linear regression calculations^56,64^. However, this limitation does not significantly impact the Random Forest algorithm, which employs bootstrapping sampling and a technique called bagging for final score computation. This method allows the algorithm to randomly select the training dataset from the whole training set with replacement and randomly selects M features or input variables from input variables. Such as methodology makes the model robust against imbalanced or skewed datasets, ensuring stable performance regardless of data standardization^19–23^.

It's crucial to understand that the results of the machine learning models in this study are specific to the dataset employed and should not be generalized. The effectiveness of each algorithm heavily depends on the characteristics of the data used, meaning Random Forest may not always outperform other algorithms in different scenarios.

**Figure 24:**
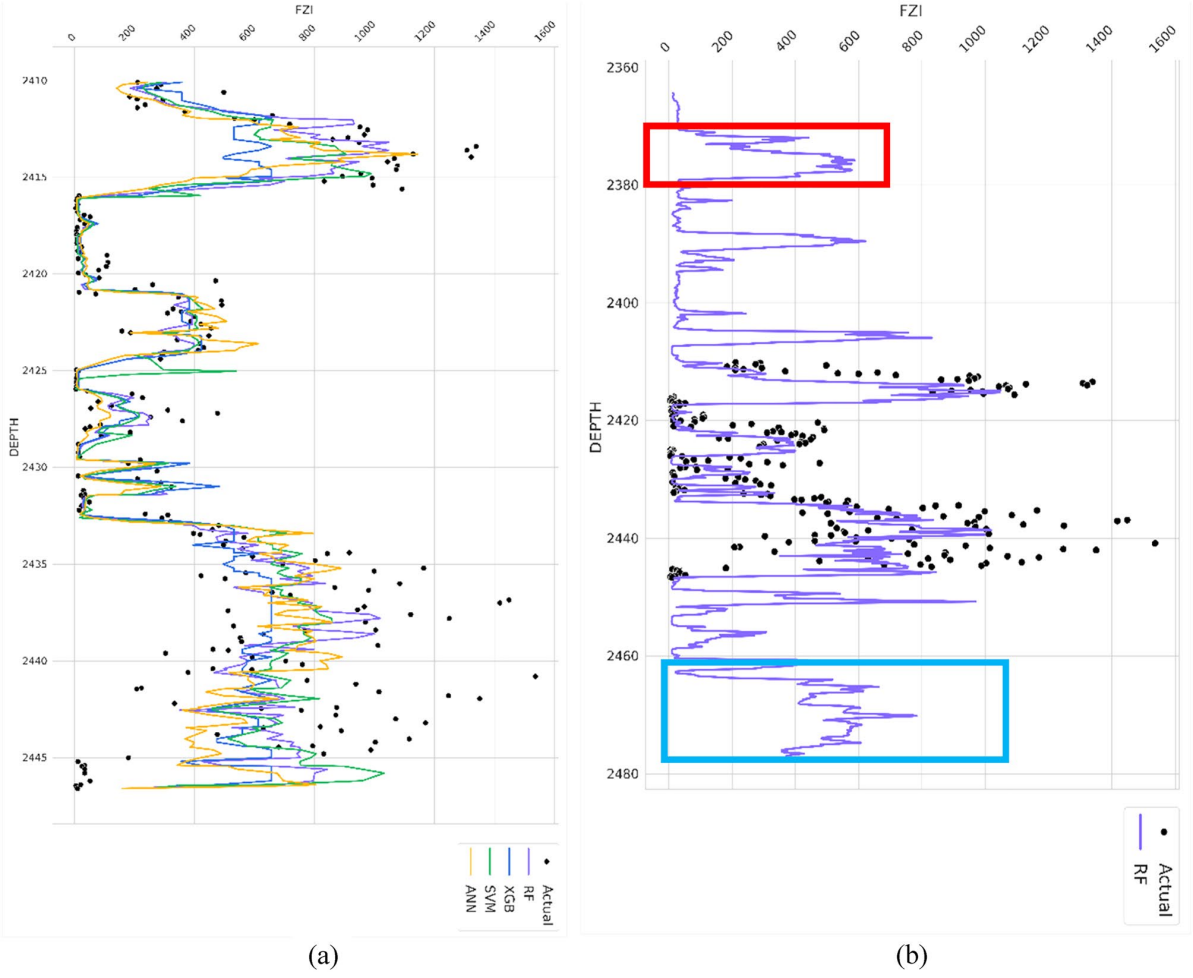
(**a**) The comparison of random forest and other algorithm performance in unseen sample data, (**b**) The prediction of FZI value using from unsampled data based on random forest model.

Figure 24 illustrates the application of the ML models in an unseen dataset, and the forecasting of FZI value using a random forest model in an unsampled location. Figure 24a reveals a noticeable resemblance between the predicted and observed trends in the FZI data. This finding holds significant value for reservoir modeling scenarios. Upon careful examination of Fig. 24b, two distinct depth ranges emerge as potential reservoir development zones. The first zone, represented by the red box, has an approximate thickness of 10 ft and an average FZI value of 500. Based on the clustering analysis presented in Table 13, it is likely associated with HFU number 8, which displays a harmonic average permeability value of 2806 millidarcy (mD). The second zone, indicated by the blue box, spans approximately 15 ft, and exhibits an average FZI value of around 800. Referring to Table 13, HFU number 9 is linked to an FZI value of around 800, suggesting the presence of a zone characterized by remarkably high permeability, measuring approximately 6410 mD. These significant findings strongly indicate the existence of favorable reservoir zones within the delineated areas. By combining the clustering analysis of HFUs and employing machine learning models to predict FZI based on well-log data, it becomes possible to estimate potential reservoir characterization zones. However, for an optimized approach to hydrocarbon recovery, it is imperative to consider additional petrophysical properties such as water and hydrocarbon saturation. Furthermore, accurate calculations of the initial hydrocarbon in place within these predicted potential zones should be incorporated into the analysis.

## Conclusion

This study utilized state-of-the-art machine learning methodologies to augment the efficacy of reservoir characterization. The supervised learning algorithms, including Random Forest (RF), Extreme Gradient Boosting (XGB), Support Vector Machines (SVM), and Artificial Neural Network (ANN), were used to predict Formation Zone Indicator (FZI) values in unsampled locations, while unsupervised learning technique of K-Means and Gaussian mixture clustering algorithm was employed to classify Hydrocarbon Flow Units (HFUs) in the reservoir. The findings of this study are summarized as follows:

- The four implemented algorithms demonstrate robust performance in estimating the flow zone indicator of the reservoir, yielding high coefficients of determination (R^2^) of 0.89 and 0.95 in the training and testing datasets, respectively.
- The RF model emerged as the optimal choice for FZI prediction in unsampled locations, with R^2^ values of 0.957 for training and 0.908 for testing.
- By combining the elbow method with K-Means clustering analysis, the study effectively delineated 10 unique HFUs. Additionally, results from the Gaussian mixture clustering corroborate the observed clustering behavior of HFUs.
- The RF model demonstrated strong performance in predicting FZI values in unsampled locations, revealing two potential reservoir development zones:
  - Zone 1 (2370 ft–2380 ft): Approximately 10 ft thick with an average FZI value of 500, associated with HFU number 8.
  - Zone 2 (2463–2477 ft): Spanning around 15 ft with an average FZI value of approximately 800, corresponding to HFU number 9 and indicating a zone characterized by remarkably high permeability.

The study's findings hold significant implications for reservoir characterization practices in the petroleum industry. The successful integration of machine learning, particularly Random Forest, into conventional methods allows for rapid and cost-effective reservoir assessments. This approach not only enhances decision-making speed but also identifies specific zones with high-quality reservoir potential. The study showcases the robustness of machine learning in petroleum engineering applications, marking a shift towards more efficient and accurate reservoir characterization. To further advance the field, future research should explore additional machine learning models and incorporate a broader set of features for a comprehensive analysis in addition to validating the results on different datasets.

## Data Availability

Most of the data are presented in the manuscript, and a detailed sample will be provided upon request through email “ahmed.ibrahim@kfupm.edu.sa”.

## List of symbols

$$k$$: Permeability in m^2^
$${\phi }_{e}$$: Effective porosity
${K}_{T}$: Pore-level effective zoning factor
${S}_{{v}_{gr}}$: Specific surface area per unit grain volume
FZI: Flow zone indicator
HFU: Hydraulic flow unit
RF: Random forest
XGB: Extreme gradient boosting
SVM: Support vector machines
ANN: Artificial neural networks
$${F}_{s}$$: Shape factor
$$\tau$$: Tortuosity
${S}_{gv}$: Surface area per unit grain in μm
${x}_{i}^{j}$: The ith pattern belonging to the jth cluster
${c}_{j}$: Centroid of the jth cluster
CGR: Corrected gamma ray
DRHO: Bulk density correction
DTC: Delta-T compressional
GR: Gamma ray
HNPO: High-resolution enhanced thermal neutron
LLD: Laterolog deep resistivity
LLHR: High-resolution laterolog resistivity
LLS: Laterolog shallow resistivity
MRES: Mud resistivity
MSFC: Micro spherically focused resistivity
NPHI: Thermal neutron porosity
NPOR: Enhanced thermal neutron porosity
POTA: Potassium concentration
RHOB: Bulk density
SP: Spontaneous potential
THOR: Thorium concentration
URAN: Uranium concentration
${x}_{{\text{norm}}}$: Normalized value with a range of values 0–1
x: Variable on the dataset while max and min refers to the maximum and minimum value of the variable
